# Trust vs. knowledge during COVID-19: the dominance of trust in promoting preventive behaviours and its role in technology acceptance in Germany and India

**DOI:** 10.1186/s12889-026-27638-0

**Published:** 2026-05-14

**Authors:** Lahari Yaddanapudi, Julia Hahn, Felix Esslinger, Miltos Ladikas

**Affiliations:** 1 Karlsruhe Institute of Technology, Institute for Technology Assessment and Systems Analysis, Karlstrasse 11, Karlsruhe, 76133 Germany; 2 Institute of Virology, Technical University of Munich, Arcisstraße 21, München, 81675 Germany; 3 Institute of Virology, Helmholtz Zentrum München – Deutsches Forschungszentrum für Gesundheit und Umwelt (GmbH), Ingolstädter Landstraße 1, Neuherberg, 85764 Germany

**Keywords:** COVID-19 pandemic, Knowledge, Trust, Preventive behaviours, Technology acceptance, Health promotion, Health policy

## Abstract

**Background:**

Adherence to COVID-19 measures depended on a range of factors, including trust in authorities, knowledge of the virus, and public perception. This study compares Germany and India to examine the association of these factors on compliance and contributes to the debate between knowledge and trust. It also explores public acceptance of virus-deactivating technologies, which could reduce reliance on individual preventive behaviours and surveillance tools, offering new insights for pandemic response and public health strategies.

**Methods:**

Data was collected through an online primary survey using stratified random sampling in India and Germany. The questionnaire consisted of five sections: socio-demographics, knowledge, trust, preventive behaviours, and technology acceptance. The data was analysed using descriptive statistics and statistical analyses of linear regression and ordinal logistic regression.

**Results:**

Almost all preventive behaviours were more frequent in India than in Germany, while German respondents had higher overall knowledge scores. The regression analysis showed no significant relationship between knowledge and trust in India. In contrast, Germany showed a weak positive association, indicating knowledge is modestly associated with trust, though other factors are also significant. The logistic regression analysis highlighted that trust was associated with all preventive behaviours during the COVID-19 pandemic in India and Germany, consistently emerging as a significant factor. While socio-demographic factors like age, education, and income also predicted some behaviours, knowledge had only a limited association. Similar results were found for the acceptance of virus-deactivating air-purification technologies in both Germany and India. Specifically, UV-light-based air purification systems, seen as a potential replacement for measures like masks and school closures, showed high acceptance: 76% in Germany and 57% in India, suggesting a likelihood of real-world implementation.

**Conclusions:**

This study adds to existing discussions on the role of knowledge, finding that trust showed a stronger association with adherence to COVID-19 measures. Comparing Germany and India, findings show that despite vast differences in healthcare systems and priorities, trust played a similarly crucial role in both contexts for preventive behaviours and acceptance of virus-deactivating technologies. Transparent communication, co-design and co-creation approaches, as well as reflexive socio-technical collaboration may support trust-building and improve public health strategies during crises.

**Supplementary Information:**

The online version contains supplementary material available at 10.1186/s12889-026-27638-0.

## Background

During the COVID-19 pandemic, public cooperation was crucial for effectively implementing preventive measures to contain the virus. Global efforts in policy and research focused on vaccine development and distribution. However, simple preventive actions – handwashing wearing masks, and physical distancing – were also promoted as vital in limiting the virus’s spread [1]. The World Health Organization (WHO) recommended these measures which were widely adopted by governments worldwide to mitigate the spread of COVID-19. Yet, as observed in prior pandemics, achieving widespread adherence to such guidelines posed significant challenges [2, 3]. Many individuals across the world disregarded these measures [4, 5], raising the critical question: why was there resistance to complying with these life-saving recommendations?

The existing literature indicates that individual determinants of preventive behaviours are multifaceted, encompassing a range of demographic, psychological, economic, and social factors [6, 7]. Studies exploring the reasons behind adherence or non-adherence to COVID-19 precautionary measures have identified several critical factors. These include risk perception, belief in the effectiveness of the measures, trust in authorities and health information, and various socio-demographic and health-related attributes [8–11].

Several studies emphasize the importance of trust in science and government [12–16] alongside effective communication [17–19] as key to improving adherence to public health measures. Some studies argue that failure to communicate the right message effectively can result in a loss of trust, economic damage, and even loss of lives [20, 21]. Others found that messages must be timely and transparent for risk communication to successfully bridge knowledge gaps and encourage behavioural adjustments during a crisis [22]. There is nevertheless an agreement that trust in the source of information, along with clear and actionable recommendations, is critical for public compliance [20, 21, 23, 24].

A pan-European survey conducted in 2020 reported that familiarity with WHO recommendations significantly correlates with adherence [18]. Since some studies suggest that increasing knowledge increases trust, we first aim to assess this relationship in our context, by examining the following research question (RQ):

- RQ1: Is higher knowledge – about the infection, prevention and cure – associated with higher trust?

This investigation is aimed at contributing to the literature on the importance of trust and knowledge in adherence to public health guidelines. While several studies support that trust in the government or information source positively influences adherence [12–15, 25], there long exists a potential gap between knowledge and action [26], and there is a gap of investigation on the relative importance of trust and knowledge [27]. This study aims to address this gap by examining the distinct and combined roles of trust and knowledge in influencing preventive behaviours during the COVID-19 pandemic by taking the example of two countries with distinct characteristics: Germany and India. At this stage, we are cautious of the results of RQ1 and would not proceed with RQ2 if any significant relationship is found in our data between knowledge and trust.

- RQ2: How are trust and knowledge associated with adherence to preventive measures and recommendations, and which of the two has a more significant association?

In the battle against the pandemic, some researchers focused on studying the science of the virus to inform preventive measures, while others worked intensively on developing vaccines. However, others concentrated on creating new technologies to combat airborne viral infections in general [28]. For example, UV-light–based “Aerobuster” technology is a portable air-cleaning system that draws in indoor air and uses a combination of heat and UV-C light to deactivate virus-containing aerosols, making the air safer to breathe in places such as classrooms, medical offices, and restaurants [29]. Microwave technology takes a different approach by using specific low-power microwave frequencies that can disrupt and deactivate airborne coronavirus particles without heating them, offering a novel but technically complex method for reducing aerosol transmission [30]. Such technology-led approaches aim to provide a unique advantage: eliminate or at least reduce the need for individual preventive behaviours to fight the spread of viruses.

However, for such technologies to succeed, public acceptance is crucial. High-profile failures such as the collapse of HPV vaccination uptake in Japan and the withdrawal of metal-on-metal hip implants demonstrate that even clinically approved healthcare technologies can fail at scale when public trust in safety, regulation, and institutional integrity erodes [31, 32]. Yet, research on technology acceptance during COVID-19 has primarily examined surveillance tools, such as contact tracing and other digital solutions [33–35]. In contrast, technologies designed to deactivate the virus in the immediate environment, such as advanced air purification systems, have received little to no attention in research on public attitudes or acceptance.

This gap in research is significant, as these technologies lack the privacy and data security concerns commonly associated with digital surveillance tools, directing at a very different set of public concerns about technological solutions. Moreover, they could reduce reliance on surveillance systems and diminish the emphasis on individual preventive behaviours altogether. Our study therefore explores public opinion on these innovative approaches, examining their potential for public acceptance. Additionally, we seek to understand if acceptance is associated with factors such as knowledge, trust, or other determinants as identified for preventive measures. Finally, we aim to investigate whether the factors driving acceptance of these technologies differ from those influencing adherence to traditional preventive behaviours. Thus, the second part of this paper addresses the research question –

- RQ3: How are trust and knowledge associated with public acceptance of virus-deactivating technologies, such as air purification systems, and how do these technologies compare to traditional preventive measures during the COVID-19 pandemic?

We seek to answer all three research questions as a comparative study between Germany and India. While government-imposed restrictions on movement affected cultural, social, scientific, and political spheres at the global level, the impact differed between countries. For instance, it was more pronounced in Low-Income Countries (LICs), where consequences included deteriorating mental health, increased physical violence, and denial of essential services such as healthcare, education, and housing [36]. Even before the pandemic, disparities in government health expenditures as a percentage of Gross Domestic Product (GDP) showed that low-income countries were already lagging far behind the rest of the world [37]. The COVID-19 pandemic further exposed the stark differences in priorities among countries with varying socio-economic conditions. As trust not only affects the impact of responses but also is affected by government responses to health crises like the recent pandemic [38], we assume varied importance placed in knowledge and trust in these distinct contexts. Therefore, our study focuses on India as an example of a Lower-Middle Income Country (LMIC) and Germany as a High-Income Country (HIC) to understand the roles of knowledge and trust in different economic settings.

Moreover, Germany and India are comparable in population, economic power and global influence. Both Germany and India have large populations, with Germany being the most populous country in the European Union (EU) and India being the most populous country globally. Also, both Germany and India play important roles on the global stage, with Germany being a key player in the EU and a leader in many international organisations, and India being a major player in the BRICS group and a key actor in international diplomacy. Finally, Germany is one of the largest economies in the world, while India is a rapidly growing economy with a large middle class – a major differentiating factor that could cause tremendous variation in health priorities and healthcare systems: Germany boasts 7.8 hospital beds and 4.5 physicians per 1,000 inhabitants, whereas India has only 1.6 beds and 0.7 physicians per 1,000 inhabitants [39, 40].

Comparing India and Germany in this study enables a better understanding the factors contributing to better preventive behaviours and possibly greater acceptance of technologies, supporting solutions for pandemics. These countries offer distinct contexts regarding knowledge levels, trust in information sources, public trust in authorities, and attitudes towards technology. By analysing these factors, the study can uncover insights with global relevance. For example, India’s diverse population and varying levels of access to information shed light on effective strategies for disseminating knowledge and building trust among culturally diverse communities. Conversely, Germany’s varied public trust in authorities and advanced technological infrastructure provide insights into ways to promote widespread acceptance of technology-based solutions. Understanding how knowledge levels and trust influence preventive behaviours and technology acceptance can guide the development of targeted public health interventions worldwide and inform policy-makers and public health officials. Further, insights into knowledge and trust can also enable better development and adoption of technologies for pandemic preparedness and response on a global scale. In our framework, adherence to preventive measures is the behavioural outcome, while knowledge and trust are explanatory factors that may vary across national contexts. Differences in socio-economic conditions, healthcare systems, and pandemic governance between Germany and India may influence how strongly knowledge and trust are associated with adherence. Comparing both countries therefore enables an assessment of how context shapes the relative importance of these factors.

From a theoretical perspective, it is important to highlight the fact that the present study is not explicitly designed to test a specific behavioural theory, as it is not grounded in such a conceptual perspective. Our approach sits at the intersection of several established models of health behaviour and technology acceptance. It reflects key constructs of the Health Belief Model (HBM), such as perceived benefits and perceived barriers, and is also informed by the attitudinal component of the Theory of Planned Behaviour (TPB). Most strongly, however, the study aligns with trust-focused frameworks such as the 3 C/5 C models, particularly with the dimension of confidence in technologies, institutions, and responsible actors. Accordingly, the aim is to examine adherence to preventive behaviours and technology acceptance by jointly considering trust-related perceptions, knowledge, and sociodemographic characteristics.

## Methods

### Data collection

Data for this study were collected through a primary survey conducted online in India and Germany in June 2023 –a key phase since vaccines were already being administered, new variants of the virus were emerging, but less severe infections were being recorded. A professional company for running surveys and questionnaires was used to approach participants since this ensured a varied group in both countries. The company has a large pool of participants to recruit for the surveys they run. A stratified random sampling method was employed to ensure representation across key demographic groups in both countries. The final sample comprised 1,000 respondents from each country, resulting in 2,000 participants (Table 1). A sample size of 1,000 participants was selected to ensure sufficient statistical power and representativeness for the study’s objectives. This sample size allows for reliable estimation of population parameters, supports subgroup analyses with adequate precision, and reduces the margin of error to an acceptable level for survey‑based research. In addition, a sample of 1,000 is commonly used in large‑scale public health and social science surveys, providing a balance between methodological rigor and practical feasibility.

**Table 1:** Socio-demographic distribution of the survey participants

Country		Germany		India	
		*N*	%	*N*	%
Total		*1000*	*100%*	*1000*	*100%*
Gender	Male	489	48.9	510	51
	Female	511	51.1	490	49
Age	18–29 Y.	166	16.6	346	34.6
	30–39 Y.	153	15.3	237	23.7
	40–49 Y.	150	15	166	16.6
	50–59 Y.	194	19.4	129	12.9
	60 + Y.	337	33.7	122	12.2
Region	North	161	16.1	230	23
	West	351	35.1	230	23
	South	290	29	250	25
	East	198	19.8	290	29
Education	Below secondary	0	0	31	3.1
	Secondary school certificate	157	15.7	25	2.5
	High school diploma	153	15.3	77	7.7
	Vocational training	389	38.9	75	7.5
	Bachelor’ s degree	141	14.1	450	45
	Master’s degree	144	14.4	342	34.2
	Doctorate degree	16	1.6		
Income group (perceived)	Higher income group	48	4.8	72	7.2
	Higher-middle income group	141	14.1	281	28.1
	Middle income group	421	42.1	521	52.1
	Lower-middle income group	267	26.7	108	10.8
	Lower income group	123	12.3	18	1.8

A subset of items related to socio-demography, own preventive behaviours, trust and use of sources of information, and trust in institutions were adapted from the World Health Organization (WHO) COVID-19 behavioural insights survey instrument [41], while additional questions on technology acceptance were developed specifically for this study (see Supplementary Information, Chapter 1). Items on “Trust” that composed the trust composite were adapted based on the thematic framework provided by the OECD trust survey instrument [42]. The German translation was done by one of the authors who is a native German speaker.

To capture key behavioural determinants identified in prior health behaviour models, including trust-related perceptions, knowledge about the technology, and individual sociodemographic characteristics, the survey consisted of five sections:

- *Socio-Economic Information*: This section captured demographic and socio-economic data, providing context for the analysis.
- *Knowledge Section*: Participants answered true/false questions about myths and facts related to coronavirus prevention, precaution, and cure. The “Knowledge score” was constructed from these items. Item difficulty was assessed using a pilot survey, where the proportion of correct responses was used to approximate difficulty levels. Based on these results, items were categorized as easier or harder and weighted accordingly when constructing the knowledge score.
- *Trust Section*: Trust-related items answered on a five-point Likert scale focused on future scenarios to assess trust in self, friends, and family regarding preventive behaviours, as well as trust in scientists and government responses to potential future health crises. These responses were aggregated to form a “Trust Composite” score.
- *Preventive Behaviours*: Participants provided responses on three types of "preventive behaviours" items answered on a five-point Likert scale of frequency—precautionary measures, personal hygiene, and social distancing—therefore reporting on the self-assessed frequency of seven WHO-recommended preventive behaviours at three different points in time: the beginning of the pandemic, after receiving the first dose of vaccination, and at the time of the survey.
- *Technology Acceptance*: We used specific scientific research topics and technology descriptions from a large-scale five-year German research project on the detection and deactivation of the SARS-CoV-2 virus in aerosols (COREARO) [28] to compare the understanding of these technologies to a proxy indicator of its acceptance – “perceived usefulness” of the technologies, both on a 5-point Likert scale. Furthermore, to assess the rationale and patterns of technology acceptance as an alternative for preventive measures, we asked participants to choose whether they would implement a prototype machine or continue to follow the measures and whether they would change this answer if the government decided to implement it widely.

### Data analysis

To prepare for data analysis, two calculations were initially carried out:

- *Knowledge Score*: The knowledge score was computed as the total number of correct answers out of 15 true or false questions. Each correct answer was awarded 1 point, resulting in a cumulative score for each participant. Additionally, the scores were analysed based on three levels of question difficulty (easy, medium, and hard) and categorized by topic:

- Function of Masks, Sanitation, and Social Distancing.
- Food and Medicine for Prevention and Cure of COVID-19.
- Scientific Properties and Spread of the Virus.

While these sub-analyses provided a nuanced understanding of knowledge patterns, the total knowledge score was used for the regression analysis.

b. *Trust Composite*: Trust-related responses were grouped into five sections based on trust in self, friends and family, scientists, government, and future health scenarios. Each section was scored separately, and the aggregate mean of these scores was calculated to derive the overall trust composite.

We used descriptive statistics for an overview of the trends of knowledge, trust, and preventive behaviours over time. Other statistical analyses for the research questions RQ1, RQ2 and RQ3 are described in their sub-sections below.

#### RQ1 – Is higher knowledge associated with higher trust?

Given prior evidence suggesting that knowledge building may enhance trust, we sought to explore whether a similar relationship is observed within our dataset. We conducted a linear regression analysis to examine this association, with the knowledge score as the independent variable and the trust composite as the dependent variable.

#### RQ2 – Association of knowledge and trust with preventive behaviours

To further investigate the factors influencing adherence to preventive behaviours, we included both socio-demographic and economic variables in the regression model. Socio-demographic factors such as age, gender, income, and education were incorporated based on existing literature highlighting their significant role in shaping health behaviours. Additionally, the economic impact of the pandemic, represented by the variable “financial status improved or worsened over the last 3 years,” was included to assess its association with adherence to preventive measures. To account for the role of knowledge and trust, we incorporated the “knowledge score”—reflecting participants’ understanding of virus spread, prevention, and cure—and the “trust composite,” which represents trust in self, others, and institutional responses to health crises. By integrating these variables into a series of ordinal logistic regression analyses (for each of the seven preventive behaviours), we aimed to identify individual and combined effects on the frequency of preventive behaviours, measured on a 5-point Likert scale. For the purpose of this analysis, we take the “current preventive behaviours”, i.e. frequency of preventive behaviours at the time of the survey as our outcome variable. Including trust and knowledge simultaneously in the models allowed for an assessment of their relative contribution to preventive behaviour while adjusting for potential confounding by age, gender, income, and other covariates. This approach is consistent with behavioural models that conceptualise health-related actions as the result of multiple interacting cognitive, attitudinal, and structural determinants.

#### RQ3 – Public acceptance of technologies

A similar series of ordinal logistic regression analyses was conducted for the two countries, focusing on public perception of two specific technologies—UV light inactivation and microwave inactivation—for virus deactivation. Public perception of the usefulness of these technologies, measured on a five-point Likert scale, was used as the dependent variable as a proxy indicator of technology acceptance. In addition to the regression analysis, descriptive statistics were qualitatively analysed to explore public preferences regarding the adoption of these technologies in public spaces. Specifically, we examined whether participants were more inclined to accept the implementation of such technologies or preferred alternative measures, such as lockdowns and school closures, to respond to future health crises.

Analyses were conducted separately for Germany and India to examine within-country associations, as formal inferential testing of between-country differences using pooled interaction models was beyond the scope of this study.

## Results

### Preventive behaviours during different phases of the pandemic

Overall, the mean frequency of all self-reported preventive behaviours was higher in India (e.g. Current frequency Mean = 3.75, SD = 0.8) than in Germany (Current frequency Mean = 2.9, SD = 1.0) during all phases of the pandemic, except self-testing (that could be the direct result of differences in the availability and/or accessibility of self-tests in the two countries). Additionally, the frequency of disinfecting surfaces was notably lower (Current mean (DS) = 2.7, SD = 1.3) than the other behaviours (e.g. Current mean (WH) = 3.5, SD = 1.2) in Germany, whereas in India, it remained consistent with the other measures (India, current mean (DS) = 3.8, SD = 1.1).The frequency of these behaviours did not change in India over the different phases from the beginning of the pandemic to the first vaccine dose and eventually at the time of the survey. However, German respondents reported a drastic decline in these frequencies from their first vaccine dose to the time of the survey – with very low frequencies of all preventive behaviours at the time of the survey.

### Knowledge, trust, and the relationship between them (RQ1)

#### Knowledge about infection, prevention and cure

Overall, respondents from Germany had a higher average knowledge score on the knowledge test (Mean = 7.28, SD = 2.98) than those from India (Mean = 6.31, SD = 2.41). However, looking closely at the questions revealed interesting insights, i.e. when the questions were analysed based on the topic: function of masks, sanitation and social distancing, food and medicine for prevention and cure of COVID-19 disease, and scientific properties and spread of the virus. This classification revealed that India scored lower on food and medicine for prevention (India: Mean = 2.42, SD = 1.87; Germany: Mean = 3.61, SD = 1.90), indicating the prevalence of anecdotes in the country that certain foods can cure the infection. Further variations in knowledge levels for both countries by socio-demographic groups are given in Supplementary Information, Tables S1.a-b.

#### Baseline trust levels

Before examining the relationship between knowledge and trust, baseline trust levels were assessed. Comparatively, the mean trust composite score was slightly higher in India [Mean = 23.8, SD = 4.3] than in Germany [Mean = 20.8, SD = 4.9]. This pattern of higher trust in India was consistent across various individual institutions and information sources, including health workers, the WHO, and the Ministry of Health (detailed descriptive statistics and figures regarding specific trust in actors and usage of information sources can be found in Supplementary Information: Table S2, Figures S1, S2a. and S2b.).

#### Association between knowledge and trust: linear regression results

Linear regression analysis (Tables 2 and 3) showed no significant relationship between knowledge and trust in India (R² = 0.001, *p* = 0.472), indicating that knowledge explained only 0.1% of the variance in trust. In contrast, Germany exhibited a weak but statistically significant positive association (R² = 0.032, *p* < 0.001), with knowledge accounting for 3.2% of the variance in trust. However, the low R² values in both countries suggest that other factors are primarily associated with trust.

**Table 2:** Results of linear regression analysis between “Knowledge score” and “Trust composite” - Correlations

Country			Knowledge Score	Trust Composite
India	Knowledge Score	Pearson Correlation	1	− 0.023
		Sig. (2-tailed)		0.472
		N	1000	1000
	Trust Composite	Pearson Correlation	− 0.023	1
		Sig. (2-tailed)	0.472	
		N	1000	1000
Germany	Knowledge Score	Pearson Correlation	1	0.180^**^
		Sig. (2-tailed)		< 0.001
		N	1000	1000
	Trust Composite	Pearson Correlation	0.180^**^	1
		Sig. (2-tailed)	< 0.001	
		N	1000	1000

**. Correlation is significant at the 0.01 level (2-tailed)

**Table 3:** Results of linear regression analysis between “Knowledge score” and “Trust composite” – Model Summary

Country	Model	*R*	*R* Square	Adjusted *R* Square	Std. Error of the Estimate
India	1	.023^a^	0.001	0.000	4.32093
Germany	1	.180^a^	0.032	0.031	4.88340

a. Predictors: (Constant), Knowledge Score

### Factors associated with adherence to preventive behaviour recommendations (RQ2)

The ordinal logistic regression analysis (summary presented in Tables 4 and 5) revealed significant factors influencing preventive behaviours during the COVID-19 pandemic, drawing comparisons between India and Germany (see Supplementary Information, Table S3.a. and S3.b. for full result tables showing all the variables included in the model). For some of the behaviours considered, socio-demographic characteristics such as age, education and income emerged as statistically significant predictors. While knowledge was associated with only a few preventive behaviours, trust consistently emerged as a notable factor associated with *all* preventive behaviours across both countries. Overall, model fit for the ordinal logistic regressions was assessed. All models were statistically significant based on likelihood ratio tests (India: LR χ²(32) = 112.98–198.34, *p* < 0.001; Germany: LR χ²(28) = 93.71–197.71, *p* < 0.001), indicating that the predictors collectively improved model fit compared to intercept-only models. Deviance goodness-of-fit tests were non-significant for all models (*p* > 0.05), suggesting adequate agreement between observed and predicted values (Tables 6 and 7).

#### Socio-demographic factors associated with preventive behaviours

The ordinal logistic regression analysis identified significant associations between various demographic factors and the frequency of the preventive behaviours “washing hands and using sanitizers” (WH), “avoiding touching face” (ATF), “disinfecting surfaces” (DS), “avoiding social events” (ASE), “staying at home from work or school” (SH), “physical distancing” (PD), and “using self-test kits” (STK), measured on a Likert scale ranging from “regularly” to “never” (see Supplementary Information, Table S3.a. and S3.b.). Gender emerged as a significant predictor in both India and Germany, influencing specific preventive behaviours. In India, gender was associated with WH, ATF, DS, SH and PD, whereas in Germany, it was linked to WH, ATF, PD, STK. Education also played a key role, though its association was greater with preventive behaviours in India than in Germany. In India, education was significantly associated with WH, ATF, DS, ASE, PD, and STK, while in Germany, it was associated only with SH. Urban or rural area of residence was another factor, particularly prominent in Germany, where it was associated with WH, ATF, DS, ASE, SH, and PD. However, in India, rural populations were underrepresented in the survey due to the limitations of the online format, which restricted access to respondents in rural areas. As such, no concrete comparisons can be deduced from this variable.

Income was found to be associated with preventive behaviours in Germany more broadly, being significantly associated with ATF, DS, ASE, PD, and STK. In India, its association was more limited, with only STK. Work-from-home possibility emerged as a shared predictor across both countries, though the specific behaviours linked varied. In India, work-from-home possibility was associated with WH, ATF, SH, and PD, while in Germany, it was associated with ASE, SH, PD, and STK.

A unique factor in India was the change in financial situation over the past three years, which was significantly associated with WH, DS, and ASE. This factor did not emerge as significant in Germany, highlighting a contextual difference in predictors between the two countries, which also reflects differences in the economic reality between them.

Additionally, we included a variable indicating whether the respondent had ever tested positive for COVID-19 (via self-test, rapid test, or RT-PCR test) to control for the impact of personal infection history on preventive behaviours.

It is important to highlight the magnitude of the effect that certain structural and socio-economic variables had on adherence. For instance, the ability to work from home significantly increased the odds of engaging in other protective behaviours, such as avoiding social events (OR = 4.150, *p* < 0.001), demonstrating how structural flexibility at work facilitates broader adherence in daily life. These large ORs underscore that while psychological factors like trust are consistent predictors, structural capacities and economic realities heavily dictated the practical ability to adhere to certain measures.

#### Association of knowledge and trust with adherence to preventive behaviours

Trust was consistently a significant and strong predictor for all seven preventive behaviours in both countries (Tables 4 and 5). In India, trust had odds ratios ranging from 1.080 for staying at home from work/school to 1.182 for washing hands and using sanitizers. In practical terms, an odds ratio of 1.182 indicated a 18.2% increase in the odds of a respondent reporting a higher frequency of washing hands for every one-unit increase in their composite trust score. This demonstrates a meaningful, compounding behavioural impact as institutional trust increases. The Wald χ² values were consistently large, ranging from 27.464 to 105.115, and all p-values were highly significant (*p* < 0.001), demonstrating a robust and statistically significant association between trust and adherence to preventive behaviours. This suggests that individuals with more trust in health authorities and institutions were more likely to engage in social distancing, handwashing, and disinfecting surfaces.

In Germany, trust was also significantly associated with all preventive behaviours, though with slightly lower odds ratios compared to India. The odds ratios ranged from 1.062 for avoiding social events to 1.098 for using self-test kits, indicating that higher trust was generally associated with an increased likelihood of engaging in preventive actions. The Wald χ² values ranged from 16.673 to 47.137, with all p-values again being highly significant (*p* < 0.001). This further underscores the role of trust in influencing preventive behaviours in Germany, with similar trends to India but slightly weaker associations.

In contrast, knowledge had a much more limited impact (Table 4 and 5). In India, knowledge was significantly associated with only one behaviour, social distancing (SH), with an odds ratio of 0.945, Wald χ² = 5.337, and p-value = 0.021. This indicates that while knowledge had a slight negative association with social distancing, its overall association was minimal compared to trust.

In Germany, knowledge had a broader, yet still weaker, impact on preventive behaviours. Knowledge was significantly associated with four out of seven behaviours:

- Disinfecting surfaces (OR = 0.925, Wald χ² = 14.442, *p* < 0.001).
- Avoiding social events (OR = 0.949, Wald χ² = 6.276, *p* = 0.012).
- Social distancing (OR = 0.933, Wald χ² = 9.607, *p* = 0.002).
- Using self-test kits (OR = 1.051, Wald χ² = 5.816, *p* = 0.016).

The odds ratios for knowledge indicate a generally weak effect, with positive and negative associations depending on the behaviour. Knowledge slightly reduced the likelihood of behaviours like disinfecting surfaces and avoiding social events (with odds ratios below 1). Still, it increased the likelihood of using self-test kits (with an odds ratio above 1). Despite the statistical significance of these associations, the overall effect of knowledge on preventive behaviours was less pronounced than that of trust.

**Table 4:** Results summary of Ordinal Logistic Regression Analysis for Frequency of Preventive Behaviours (Germany)

		Trust	Knowledge
WH			
Wald *χ2*(1)		44.747	0.635
*p*-value		**< 0.001**	0.425
Odds ratio		1.094	0.984
95% CI	Lower	1.066	0.945
	Upper	1.123	1.024
*AT*			
Wald *χ2*(1)		32.378	0.406
*p*-value		**< 0.001**	0.524
Odds ratio		1.080	0.987
95% CI	Lower	1.051	0.949
	Upper	1.108	1.027
*ASE*			
Wald *χ2*(1)		19.823	6.276
*p*-value		**< 0.001**	**0.012**
Odds ratio		1.062	0.949
95% CI	Lower	1.034	0.910
	Upper	1.090	0.989
*SH*			
Wald *χ2*(1)		16.673	9.607
*p*-value		**< 0.001**	**0.002**
Odds ratio		1.063	0.933
95% CI	Lower	1.032	0.893
	Upper	1.095	0.975
*STK*			
Wald *χ2*(1)		24.347	5.816
*p*-value		**< 0.001**	**0.016**
Odds ratio		1.069	1.051
95% CI	Lower	1.041	1.009
	Upper	1.098	1.094
*PD*			
Wald *χ2*(1)		47.137	0.363
*p*-value		**< 0.001**	0.547
Odds ratio		1.098	0.988
95% CI	Lower	1.069	0.949
	Upper	1.127	1.028
*DS*			
Wald *χ2*(1)		26.588	14.442
*p*-value		**< 0.001**	**< 0.001**
Odds ratio		1.071	0.925
95% CI	Lower	1.043	0.889
	Upper	1.099	0.963

*OR* odds ratio, *CI* confidence interval. Bold values indicate statistical significance (*p* < 0.05)

**Table 5:** Results summary of Ordinal Logistic Regression Analysis for Frequency of Preventive Behaviours (India)

		Trust	Knowledge
WH			
Wald *χ2*(1)		105.115	1.511
*p*-value		**< 0.001**	0.219
Odds ratio		1.182	1.033
95% CI	Lower	1.145	0.981
	Upper	1.220	1.087
*AT*			
Wald *χ2*(1)		99.998	1.601
*p*-value		**< 0.001**	0.206
Odds ratio		1.169	0.969
95% CI	Lower	1.134	0.922
	Upper	1.206	1.018
*ASE*			
Wald *χ2*(1)		57.540	0.675
*p*-value		**< 0.001**	0.411
Odds ratio		1.121	0.980
95% CI	Lower	1.088	0.934
	Upper	1.154	1.028
*SH*			
Wald *χ2*(1)		27.464	5.337
*p*-value		**< 0.001**	**0.021**
Odds ratio		1.080	0.945
95% CI	Lower	1.049	0.900
	Upper	1.112	0.991
*STK*			
Wald *χ2*(1)		34.570	0.816
*p*-value		**< 0.001**	0.366
Odds ratio		1.091	0.978
95% CI	Lower	1.060	0.932
	Upper	1.123	1.026
*PD*			
Wald *χ2*(1)		59.695	1.210
*p*-value		**< 0.001**	0.271
Odds ratio		1.128	0.973
95% CI	Lower	1.094	0.926
	Upper	1.163	1.022
*DS*			
Wald *χ2*(1)		69.068	0.037
*p*-value		**< 0.001**	0.848
Odds ratio		1.139	0.995
95% CI	Lower	1.105	0.947
	Upper	1.175	1.046

*OR* odds ratio, *CI* confidence interval. Bold values indicate statistical significance (*p* < 0.05)

**Table 6:** Model fit statistics for ordinal logistic regression (Germany)

Dependent Variable	Omnibus test		Goodness of fit	
	LR χ² (df)	*p*	Deviance χ² (df)	*p* (GOF)
WH	148.142 (28)	< 0.001	2873.663 (3968)	> 0.05
ATF	104.079 (28)	< 0.001	3070.072 (3968)	> 0.05
DS	151.606 (28)	< 0.001	2987.793 (3968)	> 0.05
ASE	93.705 (28)	< 0.001	2939.822 (3968)	> 0.05
SH	197.711 (28)	< 0.001	2360.233 (3968)	> 0.05
PD	153.139 (28)	< 0.001	2990.554 (3968)	> 0.05
STK	191.334 (28)	< 0.001	2985.618 (3968)	> 0.05

**Table 7:** Model fit statistics for ordinal logistic regression models (India)

Dependent Variable	Omnibus test		Goodness of fit	
	LR χ² (df)	*p*	Deviance χ² (df)	*p* (GOF)
WH	191.963 (32)	< 0.001	2300.982 (3964)	> 0.05
ATF	193.496 (32)	< 0.001	2595.896 (3964)	> 0.05
DS	164.752 (32)	< 0.001	2650.371 (3964)	> 0.05
ASE	112.979 (32)	< 0.001	2806.334 (3964)	> 0.05
SH	117.172 (32)	< 0.001	2853.225 (3964)	> 0.05
PD	133.741 (32)	< 0.001	2510.345 (3964)	> 0.05
STK	198.340 (32)	< 0.001	2972.622 (3964)	> 0.05

### Acceptance of technology for virus detection and deactivation (RQ3)

We used the technology descriptions of two technologies developed for deactivating the coronavirus in the air in the CORAERO project [28]. These were: a “device using ultraviolet (UV) light for the inactivation of coronavirus” and a “device using microwave technology to clean air from coronavirus”. The goal was to assess whether the knowledge of the technology (indicated by the “understanding” of the technology) and the “acceptance” (indicated by its “perceived usefulness”) are connected. As the descriptions suggest, these are devices that can be placed or installed in spaces to purify air from the virus, preventing its spread and thus making behaviours such as wearing masks redundant.

**Fig. 1:**
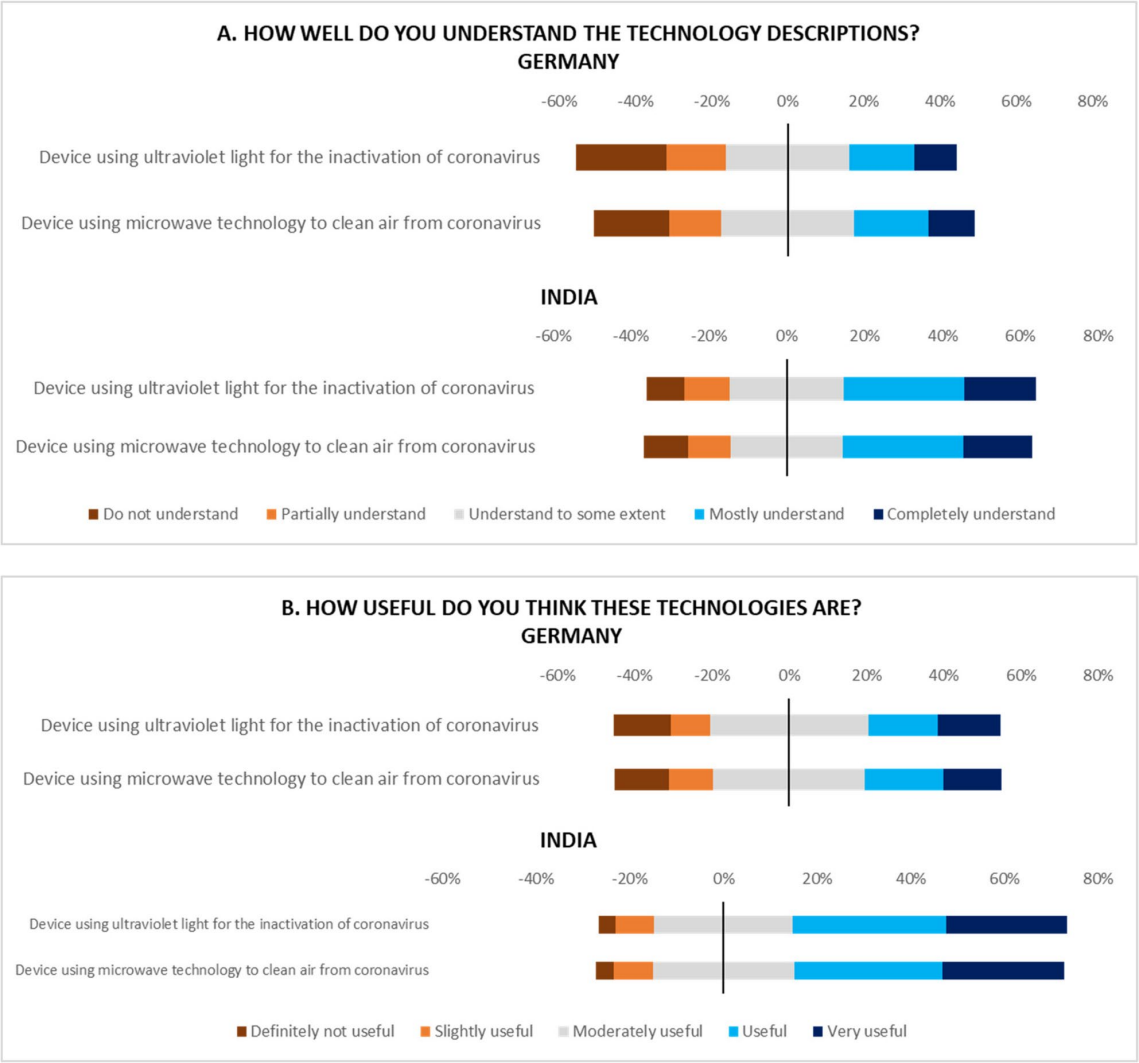
Comparison of understanding and perceived usefulness of technologies between Germany and India

Results (Fig. 1) show that in Germany, most respondents either indicate that they “mostly understand” or “completely understand” the UV light technology, with a smaller portion indicating lower levels of understanding. In India, the disclosed understanding levels are somewhat similar but slightly lower, with a notable portion only “partially understanding” or “understanding to some extent”. For the microwave technology, in Germany, understanding levels are lower compared to UV light technology. While some respondents “mostly understand,” there is a more significant portion that “does not understand” or “partially understands” the description. In India, the trend is similar to Germany, but a larger proportion of respondents indicate lower understanding, suggesting more unfamiliarity with microwave technology for cleaning the air of the virus. Therefore, there are disparities in the understanding of the two technologies.

The acceptance of UV light technology indicated by the “perceived usefulness” is also high in Germany, with the majority of respondents perceiving it as “useful” or “very useful,” and very few rating it as “slightly useful” or “definitely not useful”. The acceptance in India is also high, with most respondents marking it as “useful” or “very useful,” though there is a slightly higher proportion indicating “moderately useful” compared to Germany. However, for microwave technology, while there is some level of acceptance in Germany, a higher proportion of respondents consider it “moderately useful” rather than “very useful.” The trend is similar in India but slightly more positive, with many respondents still considering it “useful” or “very useful.” Nevertheless, both technologies have higher acceptance than their level of understanding, suggesting that even if people are not entirely familiar with the technology, they are open to its potential benefits.

Using the same indicator of acceptance of these technologies, we also found a similar pattern of trust dominance through the results of the ordinal logistic regression analysis (Tables 8 and 9). In the UV-light case, the models were statistically significant based on likelihood ratio tests (Germany: LR χ²(28) = 178.99, *p* < 0.001; India: LR χ²(32) = 173.65, *p* < 0.001), indicating that the predictors collectively improved model fit compared to intercept-only models. Deviance goodness-of-fit tests were non-significant (Germany: χ²(3968) = 2786.92, *p* > 0.05; India: χ²(3964) = 2625.70, *p* > 0.05), suggesting adequate fit between the observed and predicted values. In the microwave technology case, the ordinal logistic regression models were statistically significant in both countries (Germany: LR χ²(28) = 226.88, *p* < 0.001; India: LR χ²(32) = 190.72, *p* < 0.001), indicating that the predictors significantly improved model fit compared to intercept-only models. Deviance goodness-of-fit tests were non-significant (Germany: χ²(3968) = 2771.66, *p* > 0.05; India: χ²(3964) = 2626.92, *p* > 0.05), suggesting adequate agreement between observed and predicted values.

**Table 8:** Results of Ordinal Logistic Regression Analysis for Technology Acceptance – India

Parameter (India)	UV-light					Microwave				
	Hypothesis Test		Exp(B)	95% Wald Confidence Interval for Exp(B)		Hypothesis Test		Exp(B)	95% Wald Confidence Interval for Exp(B)	
	Wald Chi-Square	Sig.		Lower	Upper	Wald Chi-Square	Sig.		Lower	Upper
Gender										
Male	2.737	0.098	0.819	0.646	1.038	0.436	0.509	0.924	0.730	1.169
Female (ref.)			1					1		
Previous positive COVID test										
Yes	3.901	**0.048**	1.283	1.002	1.642	0.173	0.677	1.054	0.823	1.349
No (ref.)			1					1		
Vaccination status										
Yes	0.906	0.341	4.509	0.203	100.231	0.000	0.983	1.033	0.051	21.038
No	1.473	0.225	7.002	0.302	162.251	0.000	0.988	1.024	0.048	21.809
I'd rather not say (ref.)			1					1		
Education										
Literate without formal education	0.008	0.927	1.080	0.208	5.594	0.018	0.893	1.143	0.164	7.993
Below primary	1.858	0.173	0.279	0.044	1.750	2.338	0.126	0.170	0.018	1.647
Primary	1.347	0.246	0.493	0.149	1.628	2.694	0.101	0.349	0.099	1.227
Middle	0.199	0.656	1.255	0.462	3.407	0.001	0.975	1.016	0.388	2.655
Secondary	8.485	**0.004**	3.549	1.513	8.322	0.001	0.977	0.989	0.467	2.095
Higher secondary / intermediate	0.018	0.894	0.968	0.603	1.555	2.091	0.148	0.703	0.436	1.133
Non-technical diploma or certificate	0.389	0.533	1.265	0.604	2.650	0.156	0.693	1.173	0.532	2.584
Technical diploma or certificate	0.663	0.416	0.798	0.463	1.374	1.258	0.262	0.720	0.406	1.278
Graduate	0.009	0.925	1.013	0.772	1.329	0.600	0.439	0.898	0.684	1.179
Post-graduate degree and above (ref.)			1					1		
Work in the health sector										
Yes	4.153	**0.042**	1.444	1.014	2.057	5.274	**0.022**	1.513	1.063	2.155
No (ref.)			1					1		
Work from home possibility										
Yes, and I mostly work from home	0.211	0.646	0.913	0.619	1.347	1.626	0.202	1.290	0.872	1.909
Yes, and I occasionally work from home	0.000	0.983	1.004	0.684	1.475	0.270	0.603	1.108	0.753	1.631
Yes, but I don't work from home	0.530	0.467	1.171	0.765	1.793	0.024	0.877	0.967	0.630	1.485
No			1					1		
Area of residence										
Urban	0.136	0.712	0.910	0.549	1.506	4.578	**0.032**	0.574	0.345	0.954
Semi-urban	0.013	0.909	1.033	0.591	1.805	3.327	0.068	0.594	0.339	1.040
Rural (ref.)			1					1		
Household composition										
Alone	1.652	0.199	1.352	0.854	2.142	1.471	0.225	1.325	0.841	2.088
With children under 18 years of age	14.014	**<0.001**	1.869	1.347	2.593	18.216	**<0.001**	2.037	1.469	2.824
With people over 65 years and/or with chronic disease	9.644	**0.002**	1.850	1.255	2.729	2.842	0.092	1.382	0.949	2.012
None of the above (ref.)			1					1		
Income										
Higher income group	1.311	0.252	1.862	0.642	5.400	0.670	0.413	1.576	0.530	4.689
Higher-middle income group	0.086	0.770	1.157	0.436	3.073	0.044	0.835	0.899	0.330	2.446
Middle income group	0.000	0.994	0.997	0.383	2.590	0.196	0.658	0.801	0.301	2.136
Lower-middle income group	0.003	0.957	1.028	0.377	2.802	0.040	0.842	1.110	0.398	3.095
Lower income group (ref.)			1					1		
Financial situation over last 3 years										
Improved	1.017	0.313	2.710	0.390	18.822	1.700	0.192	3.600	0.525	24.695
Remained the same	0.685	0.408	2.272	0.325	15.858	1.550	0.213	3.414	0.494	23.583
Worsened	1.048	0.306	2.778	0.393	19.647	2.167	0.141	4.298	0.617	29.952
I don't know (ref.)			1					1		
Age	0.050	0.824	1.001	0.993	1.009	2.212	0.137	0.994	0.986	1.002
Trust	93.396	**<0.001**	1.158	1.124	1.193	104.299	**<0.001**	1.168	1.133	1.203
Knowledge	0.097	0.755	0.992	0.945	1.042	0.042	0.838	1.005	0.957	1.055

Note: Bold values indicate statistical significance (*p* < 0.05)

**Table 9:** Results of Ordinal Logistic Regression Analysis for Technology Acceptance - Germany

Parameter (Germany)	UV-light					Microwave				
	Hypothesis Test		Exp(B)	95% Wald Confidence Interval for Exp(B)		Hypothesis Test		Exp(B)	95% Wald Confidence Interval for Exp(B)	
	Wald Chi-Square	Sig.		Lower	Upper	Wald Chi-Square	Sig.		Lower	Upper
Gender										
Male	0.943	0.332	1.126	0.886	1.432	1.579	0.209	0.858	0.675	1.090
Female (ref.)			1					1		
Previous positive COVID test										
Yes	0.909	0.340	1.124	0.884	1.428	0.429	0.513	1.084	0.852	1.379
No (ref.)			1					1		
Vaccination status										
Yes	0.239	0.625	0.742	0.224	2.456	0.216	0.642	0.754	0.229	2.482
No	0.824	0.364	0.568	0.167	1.928	1.134	0.287	0.516	0.152	1.745
I'd rather not say (ref.)			1					1		
Education										
Secondary school certificate	1.119	0.290	0.593	0.226	1.561	0.279	0.597	1.325	0.467	3.763
High school diploma	0.926	0.336	0.624	0.239	1.631	0.219	0.640	1.281	0.454	3.612
Vocational training	0.495	0.482	0.715	0.280	1.823	0.310	0.577	1.334	0.484	3.677
Bachelor's degree	0.336	0.562	0.753	0.288	1.966	0.458	0.499	1.430	0.507	4.034
Master's degree	0.141	0.707	0.834	0.323	2.153	0.624	0.430	1.511	0.543	4.208
Doctorate degree and above (ref.)			1					1		
Work in the health sector										
Yes	0.003	0.957	1.011	0.672	1.522	0.086	0.769	1.061	0.716	1.571
No (ref.)			1					1		
Work from home possibility										
Yes, and I mostly work from home	0.782	0.377	1.175	0.821	1.682	0.099	0.753	1.059	0.742	1.511
Yes, and I occasionally work from home	2.910	0.088	1.354	0.956	1.918	2.442	0.118	1.319	0.932	1.865
Yes, but I don't work from home	0.661	0.416	0.863	0.604	1.231	0.446	0.504	0.888	0.626	1.260
No			1					1		
Area of residence										
Urban	3.180	0.075	1.295	0.975	1.720	3.957	**0.047**	1.332	1.004	1.768
Semi-urban	1.237	0.266	1.207	0.866	1.681	1.747	0.186	1.251	0.897	1.745
Rural (ref.)			1					1		
Household composition										
Alone	0.442	0.506	0.903	0.668	1.220	1.328	0.249	1.194	0.883	1.613
With children under 18 years of age	4.695	**0.030**	1.437	1.035	1.996	10.439	**0.001**	1.722	1.238	2.395
With people over 65 years and/or with chronic disease	1.235	0.266	1.235	0.851	1.793	3.736	0.053	1.441	0.995	2.086
None of the above (ref.)			1					1		
Income										
Higher income group	0.333	0.564	1.237	0.601	2.544	3.847	**0.050**	2.084	1.001	4.342
Higher-middle income group	1.948	0.163	0.694	0.415	1.159	2.691	0.101	1.530	0.920	2.544
Middle income group	0.196	0.658	0.912	0.607	1.371	1.218	0.270	1.252	0.840	1.868
Lower-middle income group	0.623	0.430	0.847	0.560	1.280	0.371	0.543	1.135	0.755	1.704
Lower income group (ref.)			1					1		
Financial situation over last 3 years										
Improved	0.000	0.993	1.005	0.356	2.831	0.361	0.548	0.735	0.269	2.007
Remained the same	0.001	0.971	0.982	0.360	2.678	0.357	0.550	0.743	0.281	1.968
Worsened	0.209	0.648	0.791	0.289	2.163	0.403	0.526	0.729	0.274	1.937
I don't know (ref.)			1					1		
Age	13.548	**<0.001**	0.984	0.976	0.993	11.864	**0.001**	0.985	0.977	0.994
Trust	50.336	**<0.001**	1.102	1.073	1.132	80.895	**<0.001**	1.132	1.102	1.163
Knowledge	3.116	0.078	1.037	0.996	1.080	2.062	0.151	1.030	0.989	1.072

Note: Bold values indicate statistical significance (*p* < 0.05)

Acceptance of the technologies in both Germany and India was not associated with knowledge but was associated with trust:

- Device using ultraviolet light for the inactivation of coronavirus (Germany: OR = 1.102, Wald χ² = 50.3, *p* < 0.001, India: OR = 1.158, Wald χ² = 93.3, *p* < 0.001).
- Device using microwave technology to clean air from the virus (Germany: OR = 1.132, Wald χ² = 80.8, *p* < 0.001, India: OR = 1.168, Wald χ² = 104.2, *p* < 0.001).

To fully answer RQ3, we also examined other socio-demographic factors influencing the acceptance of these technologies. In India, household composition played a major role. Respondents living with children under 18 years showed a strong, significant association with higher acceptance of UV light technology (OR = 1.869, *p* < 0.001), demonstrating an effect size even larger than that of trust. In Germany, higher educational attainment showed slight negative associations with UV light technology acceptance (holding a Master’s degree: OR = 0.707, though not statistically significant at *p* = 0.323). These findings indicate that while trust remains the dominant universal predictor, specific localized factors, such as protecting vulnerable household members in India, also significantly drive the acceptance of novel health technologies.

**Fig. 2:**
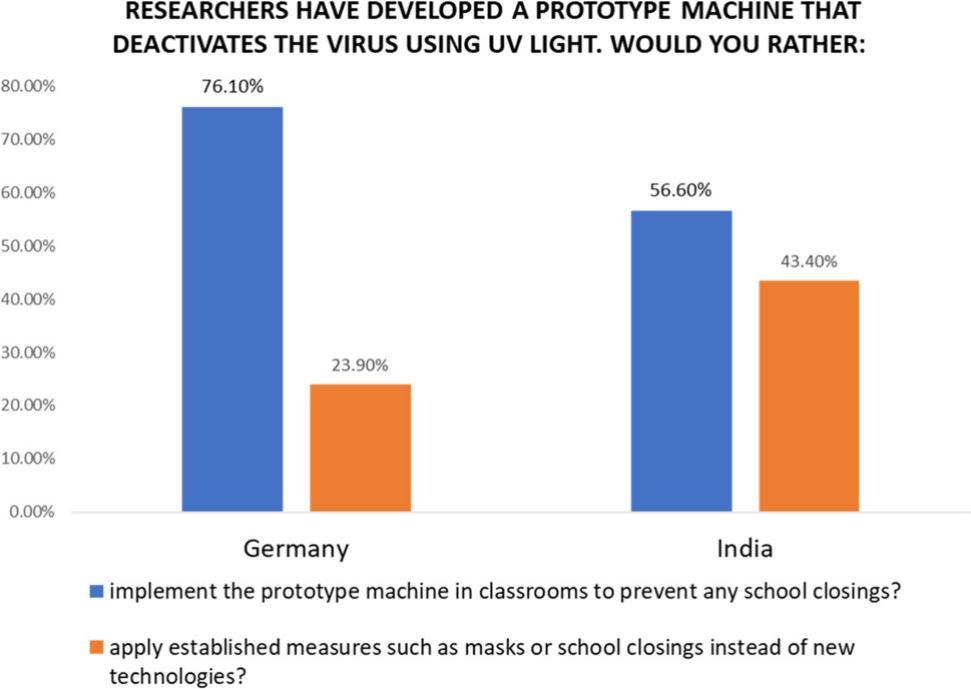
Willingness to replace established measures with UV-light technology in Germany and India

An additional question that aimed to understand the willingness of the respondents to accept the UV light technology was added. This focus arose because UV light technology had progressed further in testing and development, making it particularly relevant for providing real-time feedback to the project team. Since a main goal within the project was to provide “real-time” feedback to the researchers on their activities, this selection proved to be most useful. Therefore it was chosen to contrast towards potential replacement to measures like wearing masks and school closures. This in turn showed a similar trend of high acceptance of technologies. The findings suggest that in Germany, 76% of respondents would likely support the implementation of this technology, aligning with their higher comprehension levels and trust in scientific advancements. In India, while understanding levels were slightly lower, the technology still garnered significant acceptance, with 57% of respondents showing willingness for real-world implementation (Fig. 2). Stratifying these results by socio-demographics showed a consistent pattern of acceptance across all groups (descriptive statistics from Supplementary Information, Table S4). Since UV technology is well understood and well accepted, these figures likely align with actual implementation willingness.

## Discussion

Taken together, our findings provide clear answers to the three research questions, in our specific contexts. Regarding RQ1, knowledge was not significantly associated with trust in India and showed only a weak positive association in Germany, indicating that knowledge explains little of the variation in trust levels. For RQ2, while knowledge was associated with selected preventive behaviours, trust demonstrated a more consistent association across behaviours and countries. For RQ3, acceptance of virus-deactivating technologies was generally high despite limited public understanding, and acceptance was associated with trust rather than knowledge. Our findings thus contribute to trust-centred frameworks such as the 3 C model and 5 C model by highlighting the comparatively stronger and more consistent association of confidence-related factors with behavioural adherence and technology acceptance across national contexts, while offering empirical nuance to constructs within the Health Belief Model and the Theory of Planned Behaviour regarding the role of knowledge-based determinants.

### The dominance of trust and the limited role of knowledge

Our findings highlight the critical role of trust as a key predictor of adherence to public health measures during the COVID-19 pandemic. Across both India and Germany, trust in health authorities and institutions was consistently and strongly associated with engagement in preventive behaviours such as social distancing, handwashing, and disinfecting surfaces. These results align with prior research emphasizing the importance of trust in public institutions for effective risk communication and adherence to public health guidelines [12, 13, 18, 19]. This is also consistent with previous studies that argue that trust fosters acceptance of recommendations and mitigates scepticism about public health messages [5]. Interestingly, while the strength of this association was slightly higher in India than in Germany (as indicated by the odds ratios and Wald χ² values), the overall trend remained consistent. This variation could be attributed to contextual differences between the two countries, such as variations in government communication strategies (e.g., India’s use of mobile phone caller tunes and public address systems for mass public messaging complementing official briefings, vs. Germany’s stronger reliance on federal and state-level press conferences involving political leaders and scientific authorities), levels of institutional transparency, or public attitudes toward health authorities [38]. Further research could investigate how these contextual factors modulate the relationship between trust and adherence, particularly in culturally diverse settings.

In contrast, knowledge demonstrated a weaker and more inconsistent association on behaviour, suggesting that knowledge alone may not suffice to drive compliance. While knowledge significantly predicted certain behaviours—such as social distancing in India and four behaviours in Germany—its effects were generally weak, with some odds ratios suggesting a slight negative association. This finding contrasts with some previous studies that observed positive associations between knowledge and adherence, as well as between trust and adherence [18, 19], though the interrelationships between these factors remain complex and context-dependent. One potential explanation could be that while knowledge provides individuals with the “why” behind preventive measures, its impact is contingent on complementary factors like trust, communication quality, and social norms. Additionally, the slight negative associations observed for some behaviours (e.g., disinfecting surfaces in Germany) warrant further investigation. While our data does not directly test this explanation, individuals with higher knowledge may prioritize behaviours they perceive as most effective, potentially neglecting others. This nuance underscores the need for communication strategies that not only convey information but also contextualize the relative importance of various preventive behaviours.

While certain previous studies have examined whether knowledge dissemination is associated with adherence behaviours [10, 18], the evidence remains mixed. Our results indicate that trust shows a more consistent association with adherence than knowledge, irrespective of the behaviour or country. This suggests that trust may act as an independent and more powerful driver of compliance, even when knowledge is limited.

### Acceptance of technologies that potentially reduce reliance on individual behaviours

Today, any global challenge and crisis requires the development of technological solutions. Especially in the context of the pandemic where there was a heavy reliance on individual behaviour, these technologies could help reduce that burden. For effective implementation of these technologies, their acceptance is crucial, as demonstrated in other healthcare technologies contexts [31, 32]. Our results, which take the example of two such technologies, show that although they are not entirely understood, the public in both countries is willing to accept their implementation at the time of the survey. However, it must be acknowledged that contrasting the UV light technology specifically against school closings may inherently drive higher acceptance rates due to the strong negative societal, educational, and economic connotations associated with closures. While our survey also asked about mask-wearing in public spaces, future research must continue to evaluate technology acceptance against these less disruptive baseline measures to gauge true public willingness. Therefore, a comprehensive approach to addressing a crisis such as COVID-19 should also include approaches that take technology acceptance into account. This can help identify parameters for approving technological developments and under which conditions they are “trusted”. Moreover, trust emerged as a significant predictor of acceptance of these technological solutions, suggesting that trust may play a more consistent role than knowledge in both preventive behaviour adherence and technology acceptance. These findings highlight the importance of trust-building as a key component of health crisis management strategies. However, given the cross-sectional design on both prior studies and the present analysis, causal interpretations should be made with caution.

## Conclusion

We set up this study with the view that the relationship between knowledge and behaviour is not as straightforward as many believe. A common assumption in risk communication is that increasing knowledge leads to improved behavioural responses, although research in the social and behavioural sciences has long highlighted a potential gap between knowledge and action [26]. On the other hand, the role of trust is widely acknowledged in predicting behaviour, though fewer studies have assessed its role alongside knowledge within the same analytical framework. The triptych knowledge/trust/behaviour provides a useful lens in understanding public responses during emergencies like COVID-19. Moreover, it is vital in public health policies that strive to reduce health risks and minimise societal disruption.

The contribution of this study is threefold – firstly, our findings do not support the expectation suggested by several previous studies suggesting knowledge dissemination to build trust, with our data from both the German and Indian contexts not showing any significant relationship between the two. Secondly, we conclude from this extensive study that trust showed the most consistent association with preventive behaviours during the COVID-19 pandemic and that knowledge had only a minor role to play, thus contributing to the “knowledge” versus “trust” debate. Thirdly, we fill the gap in research on the public acceptance of technological solutions that are thought to reduce reliance on individual behaviours – finding that trust is also important for this. Additionally, the comparison between Germany and India provided a start for understanding contextual influence in the above three aspects, interestingly concluding that despite substantial differences in health priorities and healthcare systems, a strong association between trust and behavioural outcomes was observed in both contexts.

Since trust showed the strongest and most consistent association with adherence in both countries to preventive behaviours, efforts to build and sustain trust in health authorities are essential. This could include transparent and timely communication [22], consistent messaging across channels [17], and involving trusted community leaders as messengers [25]. At the same time, knowledge dissemination should not be overlooked. However, instead of focusing solely on the “what” (e.g., guidelines, such as [18]), communication strategies should also emphasize the “why” and “how” behind preventive measures, as this could enhance both trust and behavioural engagement. Although trust emerged as a dominant factor with a limited role of knowledge, certain preventive behaviours showed associations, albeit weak, with knowledge, in Germany. Therefore, future studies could investigate whether enhancing both simultaneously with contextual consideration—such as through transparent and contextually relevant messaging—can produce additive or multiplicative effects on adherence.

Furthermore, with the emergence of technologies as potential alternatives to reduce reliance on individual preventive behaviours, the finding that trust is a predominant driver of their acceptance opens unique opportunities for future health-crisis management. As the findings reported here are from a survey conducted amid the pandemic, or almost nearing its end, a similar survey at the current time could provide insights on whether this acceptance continues. Nevertheless, we emphasize the importance of incorporating these aspects early in the development of such technologies. Co-creation and co-design approaches that have been picking up momentum in the public health scene (e.g. [43]). could play a crucial role in ensuring that the perspectives and concerns of the public are integrated into the technology development process.

Additionally, the following considerations are exploratory and reflect emerging discussions in the literature. We point to the requirement of a proactive approach by engineers, scientists, and researchers that emphasizes socio-technical collaboration and ethical reflection. Integrating these elements into development processes may help technologies better align with societal values and expectations. Methods of socio-technical collaboration have been proposed as useful frameworks for pursuing this, encouraging researchers to reflect on their work’s ethical, social, and cultural implications [44] throughout the development lifecycle. Embedding such practices may contribute to making science and technology more transparent, inclusive, and responsive to public concerns, which could support perceptions of trustworthiness [45, 46]. Also, promoting ethical training and reflective practices among researchers can help them anticipate potential social impacts and navigate dilemmas with greater accountability [47, 48]. By prioritizing these efforts, engineers, scientists, and researchers may help narrow the gap between technical innovation and societal trust, and this has been discussed as a potential pathway toward improving acceptance and successful implementation of technologies for health crisis management, and public health in general.

## Supplementary Information

Supplementary Material 1.

## Data Availability

The datasets used and/or analysed during the current study are available from the corresponding author on reasonable request.

## Abbreviations

BRICS: BRICS is an intergovernmental organization consisting of ten countries—Brazil, Russia, India, China, South Africa, Egypt, Ethiopia, Indonesia, Iran and the United Arab Emirates
CORAERO: Airborne Transmission of SARS Coronavirus (project)
COVID-19: Coronavirus disease 2019
EU: European Union
GDP: Gross Domestic Product
HBM: Health Belief Model
HIC: High-Income Country
LIC: Low-Income Country
LMIC: Lower-Middle Income Country
PB: Preventive Behaviour
RQ: Research Question
STIR: Socio-Technical Integration Research
TPB: Theory of Planned Behaviour
UV: Ultra-Violet
WHO: World Health Organisation
