# Supplementary material for: Trust vs. knowledge during COVID-19: the dominance of trust in promoting preventive behaviours and its role in technology acceptance in Germany and India
Source: BMC Public Health. 2026 May 14;26:1559. doi: 10.1186/s12889-026-27638-0 (PMC13173705; doi:10.1186/s12889-026-27638-0)
Supplement: Supplementary file 1 — Supplementary Material 1. [file 12889_2026_27638_MOESM1_ESM.pdf]

SUPPLEMENTARY INFORMATION

Trust vs. Knowledge during COVID-19: The dominance of trust in promoting preventive behaviours and its role in technology acceptance in Germany and India

Chapter 1. Survey Questionnaire

KNOWLEDGE, PREVENTIVE BEHAVIOURS AND TRUST DURING COVID-19 PANDEMIC

I. Socio-demographics

1. How old are you?

<options>

2. What is your sex?
    a. Male
    b. Female
    c. Other
3. What is your education level?

<options>

4. Do you work in the Health / Medical sector?
    a. Yes
    b. No
5. Is it possible for you to work from home?
    a. Yes
    b. No
6. Where do you live?
    a. Urban area
    b. Semi-urban area
    c. Rural area
7. Which state do you live in? *[Drop down list of states]*
8. Which district do you live in? *[Drop down list of districts]*
9. What is your nationality?
    a. Germany
    b. Other EU countries

    c. Non-EU countries
10. Who lives in your household besides yourself?
    a. I live alone
    b. I live with children under 18 years
    c. I live with people over 65 years and/or with chronic disease
    d. None of the above
11. Please indicate which income strata you think you belong to
    a. Higher income group
    b. Higher-middle income group
    c. Middle income group
    d. Lower-middle income group
    e. Lower income group
12. Please assess your financial situation over the past three years:
    a. Improved
    b. Remained the same
    c. Worsened
    d. Don't know

II. Preventive Behaviours

We would like to know your own practices related to COVID-19

1. Currently, which of the following measures do you take to prevent infection from COVID-19? (1-5 scale – always, sometimes, never / very frequent to never)
    a. Frequently washed my hands with soap and water for at least 20 seconds or used disinfectants (sanitizers)
    b. Avoided touching my eyes, nose and mouth with unwashed hands
    c. Avoided a social event I wanted to attend
    d. Stayed at home from work/school
    e. Used self-testing kits whenever in doubt
    f. Ensured physical distancing in public
    g. Disinfected surfaces
2. Recall when you received your first dose of vaccination. How frequently did you take the following measures to prevent infection from COVID-19? (1-5 scale – always, sometimes, never / very frequent to never)
    a. Frequently washed my hands with soap and water for at least 20 seconds or used disinfectants (sanitizers)
    b. Avoided touching my eyes, nose and mouth with unwashed hands

c. Avoided a social event I wanted to attend
d. Stayed at home from work/school
e. Used self-testing kits whenever in doubt
f. Ensured physical distancing in public
g. Disinfected surfaces

3. Remembering when the first news about the COVID-19 pandemic outbreak in the very beginning, how frequently did you then take the following measures to prevent infection? (1-5 scale – always, sometimes, never / very frequent to never)
   a. Frequently washed my hands with soap and water for at least 20 seconds or used disinfectants (sanitizers)
   b. Avoided touching my eyes, nose and mouth with unwashed hands
   c. Avoided a social event I wanted to attend
   d. Stayed at home from work/school
   e. Used self-testing kits whenever in doubt
   f. Ensured physical distancing in public
   g. Disinfected surfaces
4. If you have been in contact with someone who tested positive for COVID-19 and have no symptoms yourself – will you get tested?
   a. I would get tested for sure
   b. I may not get tested
5. If you test positive for COVID-19 and are asked to share with health authorities the names of people you have been in contact with – will you share all names?
   a. I would share all names for sure
   b. I may not share all names

III. Trust

General

Imagine it is 2043 and a new air-borne virus outbreak is identified in Germany. Agree or disagree (1-5 scale)

1. To what extent do you agree/disagree with the following statements:
   a. I will not be worried because my government will implement necessary measures in public places to contain the spread of the virus.
   b. I will not be worried because scientists and researchers will develop a vaccine/deactivation machine against it soon.
   c. I will not be worried because my friends, neighbours and family will take necessary precautionary measures such as masks and social distancing until we are risk free.
   d. I will not be worried because I know how to protect myself.
   e. I will be worried

2. Which of the following affected factors would you be the most concerned about? (Ranking top 3)
    a. Economic prosperity
    b. Social coherence
    c. Political stability
    d. Cultural activities/heritage
    e. Educational opportunities
    f. Mental health of individuals
    g. Community activities

Drivers of Trust in Institutions

1. (Integrity)

If a potential new virus outbreak is identified before it's spread, how likely or unlikely do you think it is that (1-5 for each)

a. Scientists of your country will report it to the governments and the public
b. Government will issue a public statement/warning
c. The regulations will be free of political influence

2. (Responsiveness)

If a new virus outbreak is identified that is rapidly spreading, how likely or unlikely do you think it is that

a. Scientists will immediately convene research on the spread and containment
b. Government will immediately implement containment measures

If many people complained that testing/treatment of the new virus is inadequate, how likely or unlikely do you think it is that

a. Public health institutions will channel their resources to address the problem
b. The government would address concerns raised by the public

3. (Reliability)

If a new contagious disease spreads, how likely or unlikely do you think it is that

a. Scientific and health institutions will be willing to work overtime
b. Government institutions will be prepared to protect people's life
c. Personal data collected during testing will be used for legitimate purposes

4. (Openness)

If many measures are implemented to prevent or contain the spread of a contagious disease, how likely or unlikely do you think it is that

a. You would have an opportunity to voice your views if a measure/policy affects you or your community directly
b. Information will be easily available on prevention and treatment

5. (Fairness)

If a local community is infected by the virus and needs treatment, how likely or unlikely do you think it is that

a. The rich and the poor will be treated with equal importance and care
b. All people will be treated equally regardless of their gender, sexual identity, ethnicity or country of origin

Trust in Sources of Information

1. Do you trust information about COVID-19 from the following sources? (1-5 scale)
    a. Television
    b. Newspapers
    c. Health workers
    d. Social Media
    e. Radio
    f. Ministry of health
    g. Politicians and leaders
    h. World Health Organisation (WHO)
    i. Robert Koch Institute
    j. National COVID-19 information website
2. How often do you use the following sources for information about COVID-19? (1-5 scale)
    a. Television
    b. Newspapers
    c. Health workers
    d. Social Media
    e. Radio
    f. Ministry of health
    g. Politicians and leaders
    h. World Health Organisation (WHO)
    i. Robert Koch Institute
    j. National COVID-19 information website
3. How much confidence do you have that the following can handle the COVID-19 challenge well? (1-5 scale)
    a. Your family doctor
    b. Your employer

c. Hospitals
d. Ministry of Health
e. Schools
f. Public Transportation Companies
g. Police
h. Places of worship

IV. Knowledge

Please respond to the following in True/False to the best of your knowledge. Kindly do not guess, and select "Don't know" in case you are not sure.

1. Vitamin and mineral supplements can cure COVID-19 (F)
2. Exposing yourself to the sun or temperatures higher than 25°C protects you from COVID-19. (F)
3. Remdesivir is a drug that may shorten the recovery time for COVID-19 virus (T)
4. Cold weather and snow cannot kill the COVID-19 virus. (T)
5. The COVID-19 virus can be spread through mosquito bites. (F)
6. Hand dryers are effective in killing the COVID-19 virus. (F)
7. COVID-19 vaccines cause you to test positive for COVID-19. (F)
8. People vaccinated for COVID-19 cannot get infected. (F)
9. Transmission of active COVID-19 (virus that can cause infection) through breast milk and breastfeeding HAS NOT been detected to date. (T)
10. Eating acidic fruits cures the new coronavirus as the acids in the fruit kill it. (F)
11. COVID-19 virus is sensitive to ultraviolet (UV) light. (T)
12. Mixing different vaccines for COVID-19 is safe. (T)
13. Diarrhoea is a symptom of COVID-19. (T)
14. The range of incubation period for COVID-19 virus is 1-7 days (F)
15. COVID-19 is more contagious from asymptomatic people than symptomatic people (F)

Red → Easy (5)

Green → Medium (5)

Blue → Hard (5)

V. Acceptance of Technology (CORAERO technology)

1. Many researchers are working to develop technologies to detect and deactivate the virus in many ways. To what extend do you understand the descriptions of the following technologies: 1-5 scale

a. A simulation model predicting virus spread in rooms and public transportations
b. Non-infectious SARS-CoV-2 mimics to support research
c. Study of the physical properties of virus loaded aerosols
d. Effects of various environmental factors, such as UV light, air temperature, air humidity, and ozone concentration, on the transmission of coronavirus
e. Study of the filtration mechanism of masks
f. Study of the evaporation rate of virus from surfaces
g. How the micro-environment of saliva aerosols save and kill viruses
h. Biomarkers for improved diagnosis, prognosis, and therapeutics
i. Study of antiviral immune response in nasal cell cultures after exposure with complex bio-aerosols made up of pollen- and coronavirus particles
j. Solar inactivation of Coronavirus
k. UV-light Inactivation of Coronavirus
l. Microwaves to clean the air from viruses - no radiation or chemicals

2. Many researchers are working to develop technologies to detect and deactivate the virus in many ways. Please share your opinion on the following technologies: Very useful/Somewhat useful/Neutral/Somewhat not useful/Definitely not useful/ Don’t know
   m. A simulation model predicting virus spread in rooms and public transportations
   n. Non-infectious SARS-CoV-2 mimics to support research
   o. Study of the physical properties of virus loaded aerosols
   p. Effects of various environmental factors, such as UV light, air temperature, air humidity, and ozone concentration, on the transmission of coronavirus
   q. Study of the filtration mechanism of masks
   r. Study of the evaporation rate of virus from surfaces
   s. How the micro-environment of saliva aerosols save and kill viruses
   t. Biomarkers for improved diagnosis, prognosis, and therapeutics
   u. Study of antiviral immune response in nasal cell cultures after exposure with complex bio-aerosols made up of pollen- and coronavirus particles
   v. Solar inactivation of Coronavirus
   w. UV-light Inactivation of Coronavirus
   x. Microwaves to clean the air from viruses - no radiation or chemicals

3. Researchers have developed a prototype machine that deactivates the virus using UV light. Would you rather:
   a. implement the prototype machine in classrooms to prevent any school closings?

      OR

   b. apply established measures such as masks or school closings instead of new technologies?
4. The government backs the UV-light deactivation technology and implements it in certain public closed spaces. Would you:

a. Be mask-free and social distancing free in these public spaces?

OR

b. Avoid these public spaces or still use masks and maintain distance from people?

Just one last question!

Are you vaccinated?

Yes/No

Thank you!

Chapter 2. Supplementary Tables and Figures

Table S1.a. Descriptive statistics of knowledge scores – Germany

		KNOWLEDGE SCORE (TOTAL)		KNOWLEDGE SCORE (A)		KNOWLEDGE SCORE (B)		KNOWLEDGE SCORE (C)	
		Mean	S.D.	Mean	S.D.	Mean	S.D.	Mean	S.D.
**GERMANY**		7.28	2.98	3.61	1.90	2.66	1.33	2.82	1.39
**AGE**									
	18-24 years	6.58	2.59	3.02	1.69	2.38	1.20	2.70	1.34
	25-54 years	7.20	3.09	3.40	1.89	2.68	1.39	2.82	1.44
	55-64 years	7.47	2.87	3.88	1.90	2.73	1.22	2.80	1.42
	65 and above	7.42	2.89	3.91	1.91	2.61	1.32	2.86	1.29
**GENDER**									
	Female	7.35	2.98	3.63	1.86	2.67	1.31	2.86	1.43
	Male	7.20	2.97	3.58	1.95	2.64	1.35	2.77	1.35
**POSITIVE COVID TEST**									
	No	7.27	2.97	3.62	1.90	2.70	1.32	2.76	1.37
	Yes	7.29	2.98	3.60	1.91	2.62	1.34	2.87	1.41
**VACCINATED**									
	I'd rather not say	5.50	2.99	2.00	2.11	2.40	1.43	2.10	0.99
	No	5.75	2.92	2.84	2.00	2.31	1.40	2.02	1.16
	Yes	7.54	2.91	3.74	1.85	2.71	1.31	2.95	1.38
**WORKING IN HEALTH SECTOR**									
	No	7.26	2.95	3.60	1.91	2.65	1.32	2.81	1.37
	Yes	7.43	3.23	3.66	1.88	2.73	1.43	2.87	1.56
**WORK FROM HOME POSSIBILITY**									
	No	7.15	3.10	3.59	1.93	2.57	1.35	2.78	1.41
	Yes, and I mostly work from home	7.37	2.83	3.66	1.91	2.65	1.24	2.88	1.36
	Yes, and I occasionally work from home	7.78	2.61	3.64	1.80	2.99	1.21	2.97	1.35
	Yes, but I do not work from home	6.99	3.08	3.56	1.95	2.50	1.44	2.70	1.40
**AREA OF RESIDENCE**									
	Rural area	7.10	3.12	3.54	1.99	2.61	1.37	2.72	1.39
	Semi-urban area	7.31	2.87	3.67	1.85	2.62	1.31	2.85	1.40
	Urban area	7.36	2.94	3.61	1.89	2.69	1.32	2.86	1.38
**HOUSEHOLD COMPOSITION**									

	living alone	7.21	2.92	3.72	1.90	2.63	1.30	2.72	1.33
	none of the above	7.10	3.17	3.44	1.99	2.54	1.30	2.84	1.48
	with children under 18 years	7.50	2.81	3.48	1.77	2.96	1.32	2.81	1.40
	with people over 65 years and/or with chronic diseases	7.41	2.93	3.88	1.92	2.51	1.39	2.96	1.31
**INCOME GROUP (PERCEIVED)**									
	Higher income group	8.50	2.02	3.75	1.73	3.65	1.08	2.98	1.10
	Higher-middle income group	7.57	3.01	3.72	1.73	2.73	1.32	2.98	1.47
	Lower income group	7.27	2.87	3.61	1.94	2.63	1.31	2.83	1.32
	Lower-middle income group	7.33	2.80	3.67	1.89	2.65	1.26	2.85	1.29
	Middle income group	7.01	3.16	3.51	1.98	2.53	1.37	2.72	1.47
**FINANCIAL SITUATION OVER THE LAST 3 YEARS**									
	Don't know	4.79	3.79	2.00	2.48	1.79	1.63	2.00	1.30
	Improved	7.82	2.75	3.62	1.76	2.92	1.36	3.08	1.37
	Remained the same	7.18	3.09	3.60	1.94	2.58	1.33	2.80	1.45
	Worsened	7.26	2.84	3.67	1.88	2.67	1.28	2.76	1.32

A = food & medicine, B = properties and spread of the virus, C = preventive measures and vaccines

Table S1.b. Descriptive statistics of knowledge scores – India

		KNOWLEDGE SCORE (TOTAL)		KNOWLEDGE SCORE (A)		KNOWLEDGE SCORE (B)		KNOWLEDGE SCORE (C)	
		**Mean**	**S.D.**	**Mean**	**S.D.**	**Mean**	**S.D.**	**Mean**	**S.D.**
**INDIA**		6.31	2.41	2.42	1.87	2.73	1.23	2.38	1.23
**AGE**									
	18-24 years	5.84	2.31	2.10	1.67	2.57	1.28	2.23	1.10
	25-54 years	6.35	2.35	2.42	1.85	2.75	1.21	2.39	1.24
	55-64 years	6.99	2.77	3.22	2.05	2.86	1.22	2.52	1.25
	65 and above	6.35	2.52	2.25	2.07	2.76	1.17	2.46	1.37
**GENDER**									
	Female	6.32	2.39	2.36	1.89	2.74	1.19	2.40	1.22
	Male	6.30	2.44	2.48	1.85	2.71	1.26	2.35	1.24
**POSITIVE COVID TEST**									
	No	6.18	2.53	2.35	1.94	2.65	1.24	2.35	1.26
	Yes	6.46	2.27	2.50	1.79	2.81	1.20	2.40	1.19
**VACCINATED**									
	No	6.25	2.64	2.04	1.98	2.77	1.35	2.45	1.15
	Yes	6.31	2.40	2.44	1.87	2.72	1.22	2.37	1.23
**WORKING IN HEALTH SECTOR**									
	No	6.26	2.46	2.41	1.90	2.68	1.23	2.38	1.25
	Yes	6.58	2.10	2.48	1.69	2.99	1.16	2.35	1.07
**WORK FROM HOME POSSIBILITY**									
	No	6.44	2.73	2.43	2.01	2.78	1.34	2.45	1.29
	Yes, and I mostly work from home	6.08	2.46	2.38	1.82	2.66	1.25	2.23	1.19
	Yes, and I occasionally work from home	6.59	2.33	2.55	1.87	2.80	1.19	2.52	1.26
	Yes, but I do not work from home	6.02	2.18	2.20	1.84	2.65	1.18	2.28	1.14
**AREA OF RESIDENCE**									
	Rural area	5.86	2.44	2.16	1.73	2.71	1.46	2.06	1.09
	Semi-urban area	5.79	2.50	2.18	1.81	2.53	1.26	2.18	1.24
	Urban area	6.45	2.38	2.49	1.89	2.77	1.20	2.44	1.23
**HOUSEHOLD COMPOSITION**									
	living alone	6.31	2.08	2.47	1.70	2.78	1.16	2.29	1.15
	none of the above	6.01	2.57	2.28	1.98	2.53	1.27	2.34	1.15
	with children under 18 years	6.45	2.39	2.50	1.84	2.77	1.23	2.42	1.26
	with people over 65 years and/or with chronic	6.25	2.49	2.30	1.95	2.76	1.19	2.34	1.25

	diseases								
**INCOME GROUP (PERCEIVED)**									
	Higher income group	6.71	2.19	2.61	1.83	3.14	1.05	2.26	1.22
	Higher-middle income group	6.35	2.28	2.48	1.78	2.72	1.21	2.40	1.21
	Lower income group	4.39	2.52	1.11	1.57	1.89	1.64	1.94	0.94
	Lower-middle income group	6.11	2.42	2.11	1.95	2.76	1.34	2.30	1.11
	Middle income group	6.34	2.48	2.47	1.91	2.69	1.20	2.41	1.26
**FINANACIAL SITUATION OVER THE LAST 3 YEARS**									
	Don't know	7.67	3.79	3.33	2.31	3.33	0.58	2.67	2.08
	Improved	6.44	2.27	2.44	1.74	2.85	1.20	2.37	1.21
	Remained the same	6.02	2.54	2.34	1.98	2.47	1.24	2.38	1.27
	Worsened	6.68	2.48	2.58	2.05	3.02	1.15	2.37	1.12
	A = food & medicine, B = properties and spread of the virus, C = preventive measures and vaccines								

Table S2. Descriptive statistics of Trust

		Germany	India
Trust composite			
	**Mean**	20.84	23.87
	**SD**	4.96	4.32
Trust in information sources: Television			
	**Mean**	3.09	3.77
	**SD**	1.20	1.04
Trust in information sources: Newspapers			
	**Mean**	2.99	3.86
	**SD**	1.17	0.99
Trust in information sources: Health workers			
	**Mean**	3.52	4.04
	**SD**	1.09	0.92
Trust in information sources: Social media			
	**Mean**	2.28	3.39
	**SD**	1.18	1.12
Trust in information sources: Radio			
	**Mean**	3.12	3.42
	**SD**	1.15	1.13
Trust in information sources: ministry of health			
	**Mean**	3.31	4.04
	**SD**	1.25	1.01
Trust in information sources: Politicians and leaders			
	**Mean**	2.57	2.87
	**SD**	1.22	1.25
Trust in information sources: World Health Organisation (WHO)			
	**Mean**	3.36	4.16
	**SD**	1.29	1.00
Trust in information sources:National COVID-19 website			
	**Mean**	3.31	4.19
	**SD**	1.23	0.92
Trust in information sources: Robert Koch Institute			
	**Mean**	3.57	
	**SD**	1.29	

Figure S1. Trust in information sources for pandemic-related information

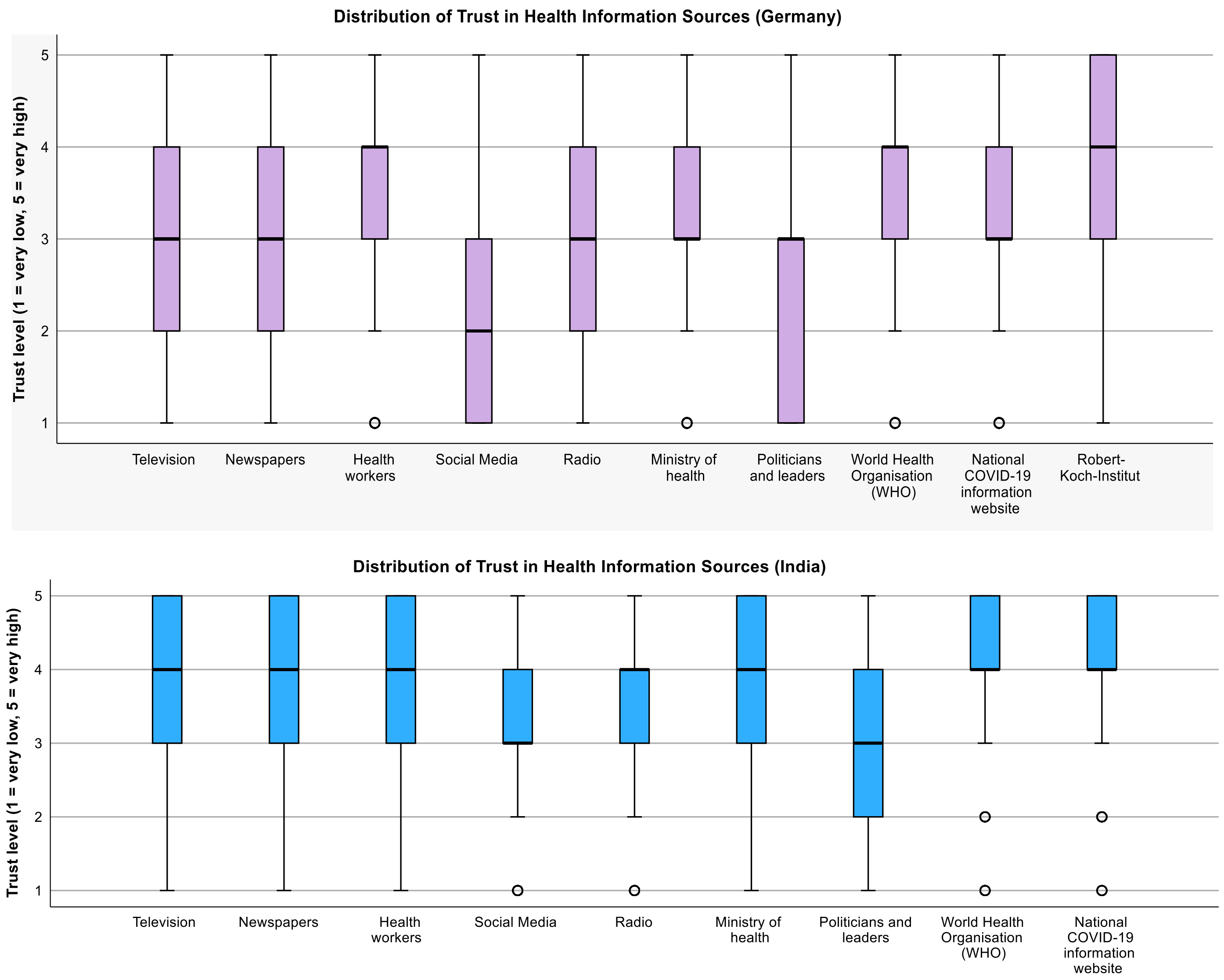

Figure S2.a. Usage of sources for COVID-19-related information in Germany

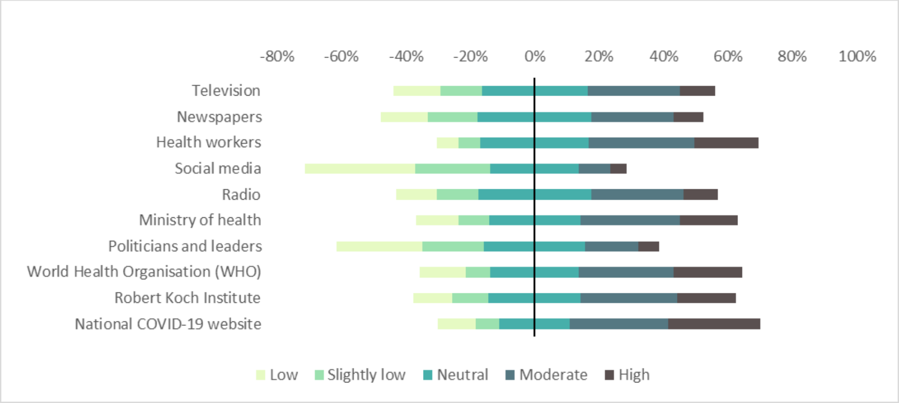

Figure S2.b. Usage of sources for COVID-19-related information in India

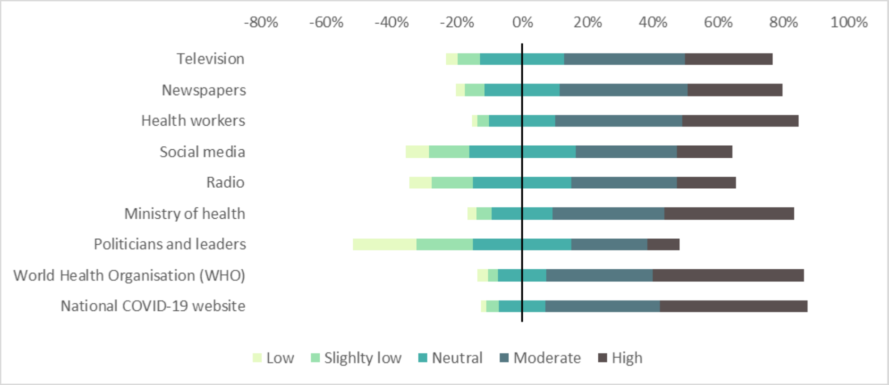

In Germany, health workers and the national COVID-19 website are the most frequently used sources of information for pandemic-related information, with 52.8% and 59.1% of respondents highly and moderately relying on them, respectively. In contrast, social media, as well as politicians and leaders, are less frequently used, with lower percentages of usage in the “high” and “moderate” categories.

In India, health workers and the national COVID-19 website, the WHO and the Ministry of Health are all dominant sources of information about the pandemic, with 74.7%, 80.2%, 79.1% and 73.9% of people using it, respectively. Additionally, traditional sources like newspapers and television are also popular, with 68.3% and 63.8% of respondents using them often. Similar to Germany, social media and politicians and leaders hold lesser usage in India as well, along with the radio (Figure S2.a. and S2.b.).

Table S3.a. Results of Ordinal Logistic Regression Analysis for Frequency of Preventive Behaviours for Germany

Parameter (India)	WH				AT				ASE				SH				STK				PD				DS			
			95% CI				95% CI				95% CI				95% CI				95% CI				95% CI				95% CI	
	p-value	Odds ratio	Lower	Upper	p-value	Odds ratio	Lower	Upper	p-value	Odds ratio	Lower	Upper	p-value	Odds ratio	Lower	Upper	p-value	Odds ratio	Lower	Upper	p-value	Odds ratio	Lower	Upper	p-value	Odds ratio	Lower	Upper
**Gender**																												
Male	**0.006**	0.706	0.550	0.905	**0.003**	0.694	0.547	0.882	0.084	0.812	0.641	1.028	**0.011**	0.739	0.584	0.933	0.187	0.856	0.680	1.078	**0.003**	0.691	0.543	0.880	**<0.001**	0.579	0.455	0.736
Female (ref.)		1				1				1				1				1				1				1		
**Previous positive COVID test**																												
Yes	0.193	0.842	0.650	1.091	0.366	0.891	0.693	1.145	0.881	0.981	0.768	1.254	0.932	1.011	0.791	1.292	**<0.001**	1.784	1.397	2.278	**0.033**	0.759	0.590	0.978	0.470	0.912	0.712	1.170
No (ref.)		1				1				1				1				1				1				1		
**Vaccination status**																												
Yes	0.638	2.062	0.101	42.014	0.769	1.561	0.080	30.610	0.999	5.052E-10	0.000	.[b]	0.999	4.495E-10	0.000	.[b]	0.999	4.295E-10	0.000	.[b]	0.965	1.070	0.055	20.783	0.999	4.347E-10	0.000	.[b]
No	0.761	1.607	0.076	34.189	0.747	1.643	0.080	33.568	0.999	3.649E-10	0.000	.[b]	0.999	3.560E-10	0.000	.[b]	0.999	4.348E-10	0.000	.[b]	0.881	0.795	0.039	16.057	0.999	3.667E-10	0.000	.[b]
??? (ref.)		1				1				1				1				1				1				1		
**Education**																												
Literate without formal education	0.943	1.067	0.177	6.430	**0.043**	0.184	0.036	0.948	0.969	0.965	0.156	5.953	0.892	1.133	0.187	6.851	0.211	0.337	0.061	1.852	0.176	0.298	0.051	1.722	0.064	0.192	0.033	1.102
Below primary	0.722	0.682	0.083	5.620	0.526	0.538	0.079	3.650	0.151	0.236	0.033	1.696	0.644	0.614	0.078	4.854	0.464	2.242	0.258	19.484	0.488	0.459	0.051	4.162	0.329	0.399	0.063	2.522
Primary	**0.009**	0.208	0.064	0.680	0.607	0.749	0.248	2.259	0.284	0.544	0.179	1.655	0.052	0.330	0.108	1.009	0.883	1.089	0.348	3.410	**0.009**	0.224	0.073	0.693	**0.049**	0.325	0.106	0.996
Middle	**0.002**	0.238	0.094	0.600	**0.014**	0.319	0.128	0.796	0.205	0.558	0.227	1.374	0.072	0.440	0.180	1.077	0.928	0.957	0.363	2.522	**0.010**	0.301	0.121	0.748	0.351	0.649	0.261	1.611
Secondary	0.450	0.744	0.346	1.601	0.062	0.500	0.241	1.036	0.163	0.580	0.270	1.246	0.626	1.204	0.570	2.541	0.460	0.760	0.366	1.576	0.198	0.591	0.266	1.316	0.121	0.548	0.256	1.173
Higher secondary / intermediate	0.401	0.805	0.484	1.336	0.055	0.617	0.377	1.010	0.457	0.829	0.506	1.359	0.974	1.009	0.610	1.668	**0.005**	0.505	0.314	0.813	0.678	0.898	0.539	1.495	0.207	0.722	0.436	1.197
Non-technical diploma or certificate	0.768	1.129	0.505	2.521	0.139	0.575	0.276	1.198	**0.015**	2.892	1.232	6.790	0.067	2.080	0.951	4.550	0.190	1.768	0.754	4.145	0.796	0.904	0.420	1.946	0.359	1.447	0.657	3.187
Technical diploma or certificate	0.277	0.726	0.408	1.293	**0.005**	0.441	0.249	0.782	0.143	0.655	0.372	1.154	0.062	0.592	0.341	1.027	0.064	0.588	0.336	1.031	0.126	0.652	0.376	1.128	**0.003**	0.445	0.258	0.766
Graduate	0.947	0.990	0.743	1.319	**0.022**	0.723	0.549	0.954	0.406	0.892	0.680	1.168	0.768	0.960	0.733	1.257	0.077	0.788	0.606	1.026	0.246	0.847	0.641	1.121	0.197	0.834	0.634	1.098
Post-graduate degree and above (ref.)		1				1				1				1				1				1				1		
**Work in the health sector**																												
Yes	0.939	1.014	0.705	1.459	0.106	1.348	0.938	1.937	0.155	1.292	0.908	1.840	0.246	1.232	0.866	1.754	**0.001**	1.793	1.261	2.550	**0.020**	1.548	1.072	2.235	**0.018**	1.548	1.077	2.224
No (ref.)		1				1				1				1				1				1				1		
**Work from home possibility**																												
Yes, and I mostly work from home	**0.021**	1.607	1.073	2.408	**0.002**	1.908	1.278	2.848	0.618	1.105	0.747	1.635	**0.003**	1.814	1.220	2.697	0.935	1.016	0.687	1.504	**0.020**	1.603	1.076	2.387	0.213	1.280	0.868	1.887
Yes, and I occasionally work from home	**0.034**	1.534	1.033	2.276	**0.015**	1.624	1.097	2.406	0.440	0.859	0.585	1.263	0.313	1.222	0.828	1.805	0.555	1.123	0.764	1.652	**0.015**	1.623	1.097	2.400	0.305	1.223	0.833	1.795
Yes, but I don't work from home	0.120	1.422	0.912	2.215	0.223	1.310	0.849	2.022	0.674	1.096	0.714	1.685	0.817	0.950	0.617	1.463	0.669	1.099	0.714	1.691	0.621	1.115	0.724	1.717	0.393	0.831	0.543	1.271
No		1				1				1				1				1				1				1		
**Area of residence**																												
Urban	0.108	0.645	0.378	1.101	0.682	1.112	0.669	1.849	0.837	1.054	0.641	1.731	0.611	0.876	0.525	1.460	0.934	0.980	0.601	1.597	0.643	0.883	0.522	1.495	**0.005**	2.084	1.255	3.459
Semi-urban	0.554	0.837	0.464	1.509	0.261	1.382	0.786	2.429	0.980	1.007	0.584	1.738	0.820	1.067	0.609	1.871	0.271	0.737	0.428	1.269	0.815	0.933	0.524	1.662	0.092	1.611	0.925	2.807
Rural (ref.)		1				1				1				1				1				1				1		
**Household composition**																												
Alone	0.631	1.125	0.695	1.824	0.448	1.198	0.752	1.908	0.638	1.116	0.707	1.761	0.842	1.048	0.661	1.662	**0.006**	1.847	1.188	2.872	0.611	1.134	0.698	1.842	0.287	0.780	0.494	1.233
With children under 18 years of age	0.740	1.060	0.751	1.496	0.156	1.269	0.913	1.766	0.869	1.028	0.737	1.435	**0.045**	0.718	0.519	0.993	**<0.001**	1.949	1.408	2.700	0.132	0.773	0.553	1.081	0.697	0.937	0.675	1.301
With people over 65 years and/or with chronic disease	0.537	1.137	0.756	1.712	0.103	1.382	0.936	2.040	0.076	1.434	0.963	2.135	0.762	0.942	0.642	1.384	**0.015**	1.621	1.099	2.391	0.923	1.020	0.683	1.523	0.617	0.906	0.615	1.334
None of the above (ref.)		1				1				1				1				1				1				1		
**Income**																												
Higher income group	0.707	1.245	0.398	3.891	0.586	0.733	0.240	2.240	0.142	2.140	0.775	5.908	0.128	2.186	0.799	5.980	**0.015**	3.746	1.291	10.869	0.489	1.476	0.490	4.449	0.936	0.959	0.345	2.664
Higher-middle income group	0.404	1.572	0.543	4.547	0.496	0.697	0.246	1.972	0.075	2.355	0.919	6.039	0.199	1.834	0.727	4.631	**0.012**	3.586	1.322	9.727	0.637	1.273	0.467	3.468	0.861	1.088	0.425	2.785
Middle income group	0.729	1.201	0.425	3.395	0.461	0.681	0.245	1.891	0.098	2.170	0.866	5.436	0.305	1.604	0.651	3.952	**0.024**	3.080	1.159	8.187	0.672	1.236	0.464	3.296	0.818	1.114	0.446	2.782
Lower-middle income group	0.605	1.333	0.449	3.960	0.264	0.545	0.187	1.582	0.133	2.095	0.799	5.491	0.485	1.403	0.542	3.627	0.158	2.093	0.751	5.833	0.655	1.263	0.453	3.518	0.913	1.055	0.402	2.767
Lower income group (ref.)		1				1				1				1				1				1				1		
**Financial situation over last 3 years**																												
Improved	**0.023**	14.232	1.443	140.332	0.167	4.234	0.548	32.717	**0.024**	11.675	1.380	98.799	**0.014**	32.731	2.028	528.184	0.242	3.638	0.419	31.592	0.335	2.533	0.383	16.733	0.413	2.172	0.338	13.937
Remained the same	**0.023**	14.507	1.458	144.325	0.152	4.490	0.575	35.034	**0.018**	13.230	1.548	113.087	**0.009**	41.787	2.568	680.073	0.246	3.620	0.413	31.773	0.265	2.942	0.442	19.598	0.252	2.982	0.461	19.314
Worsened	**0.007**	23.636	2.347	238.057	0.153	4.500	0.573	35.371	**0.010**	17.380	2.007	150.479	**0.006**	50.107	3.061	820.348	0.427	2.420	0.273	21.473	0.165	3.870	0.572	26.182	0.163	3.802	0.581	24.868
I don't know (ref.)		1				1				1				1				1				1				1		
**Age**	0.179	1.006	0.997	1.015	0.768	1.001	0.993	1.010	0.563	0.998	0.989	1.006	**0.003**	0.988	0.980	0.996	**<0.001**	0.983	0.975	0.991	0.331	0.996	0.987	1.004	0.297	1.004	0.996	1.013
**Trust**	**<0.001**	1.182	1.145	1.220	**<0.001**	1.169	1.134	1.206	**<0.001**	1.121	1.088	1.154	**<0.001**	1.080	1.049	1.112	**<0.001**	1.091	1.060	1.123	**<0.001**	1.128	1.094	1.163	**<0.001**	1.139	1.105	1.175
**Knowledge**	0.219	1.033	0.981	1.087	0.206	0.969	0.922	1.018	0.411	0.980	0.934	1.028	**0.021**	0.945	0.900	0.991	0.366	0.978	0.932	1.026	0.271	0.973	0.926	1.022	0.848	0.995	0.947	1.046

Table S3.b. Results of Ordinal Logistic Regression Analysis for Frequency of Preventive Behaviours for India

Parameter (Germany)	WH				AT				ASE				SH				STK				PD				DS			
	p-value	Odds ratio	95% CI Lower	Upper	p-value	Odds ratio	95% CI Lower	Upper	p-value	Odds ratio	95% CI Lower	Upper	p-value	Odds ratio	95% CI Lower	Upper	p-value	Odds ratio	95% CI Lower	Upper	p-value	Odds ratio	95% CI Lower	Upper	p-value	Odds ratio	95% CI Lower	Upper
**Gender**																												
Male	**0.020**	0.752	0.591	0.956	**0.011**	0.736	0.581	0.933	0.925	1.011	0.799	1.281	0.931	0.989	0.761	1.284	**0.002**	0.681	0.537	0.864	**0.004**	0.704	0.556	0.892	0.258	0.872	0.687	1.106
Female (ref.)		1				1				1				1				1				1				1		
**Previous positive COVID test**																												
Yes	0.997	1.000	0.787	1.269	0.308	0.885	0.701	1.119	0.839	1.025	0.809	1.298	0.862	1.024	0.786	1.333	**<0.001**	1.820	1.435	2.308	0.141	0.838	0.663	1.060	0.946	1.008	0.797	1.276
No (ref.)		1				1				1				1				1				1				1		
**Vaccination status**																												
Yes	0.173	2.450	0.675	8.886	0.191	2.408	0.646	8.978	0.561	0.699	0.209	2.339	0.791	1.178	0.349	3.973	0.742	1.207	0.394	3.699	0.250	2.089	0.595	7.333	0.433	1.618	0.486	5.388
No	0.953	0.961	0.259	3.574	0.527	1.540	0.404	5.869	0.145	0.398	0.116	1.374	0.692	0.776	0.221	2.724	0.125	0.405	0.128	1.285	0.964	1.030	0.287	3.700	0.883	0.912	0.267	3.114
??? (ref.)		1				1				1				1				1				1				1		
**Education**																												
Secondary school certificate	0.406	1.548	0.552	4.338	0.905	1.062	0.393	2.874	0.493	1.394	0.539	3.603	0.065	2.906	0.935	9.035	0.393	1.524	0.579	4.009	0.501	1.383	0.538	3.557	0.052	2.719	0.990	7.469
High school diploma	0.217	1.911	0.683	5.346	0.581	1.324	0.489	3.584	0.303	1.643	0.638	4.232	0.078	2.760	0.893	8.531	0.871	1.083	0.412	2.845	0.443	1.445	0.563	3.708	0.059	2.641	0.964	7.234
Vocational training	0.225	1.864	0.681	5.100	0.778	1.150	0.436	3.034	0.238	1.742	0.693	4.382	**0.039**	3.219	1.062	9.756	0.666	1.231	0.480	3.158	0.311	1.607	0.642	4.021	0.127	2.153	0.804	5.764
Bachelor's degree	0.159	2.102	0.747	5.913	0.609	1.297	0.479	3.508	0.292	1.666	0.645	4.303	0.057	2.996	0.970	9.260	0.403	1.511	0.574	3.978	0.346	1.570	0.614	4.015	0.061	2.637	0.957	7.265
Master's degree	0.425	1.513	0.547	4.186	0.884	1.076	0.403	2.869	0.468	1.414	0.555	3.601	**0.043**	3.160	1.035	9.649	0.407	1.496	0.577	3.882	0.490	1.388	0.548	3.517	0.167	2.022	0.745	5.490
Doctorate degree and above (ref.)		1				1				1				1				1				1				1		
**Work in the health sector**																												
Yes	0.383	1.197	0.799	1.793	0.211	1.297	0.863	1.948	0.676	1.091	0.726	1.639	0.546	1.146	0.737	1.781	0.882	1.031	0.692	1.534	0.762	1.063	0.717	1.575	**0.012**	1.686	1.124	2.529
No (ref.)		1				1				1				1				1				1				1		
**Work from home possibility**																												
Yes, and I mostly work from home	0.306	1.209	0.841	1.738	0.581	1.103	0.779	1.560	**0.001**	1.824	1.285	2.590	**<0.001**	6.093	4.150	8.947	0.358	1.181	0.829	1.682	**0.035**	1.450	1.026	2.050	0.570	0.905	0.641	1.277
Yes, and I occasionally work from home	0.208	0.803	0.571	1.130	0.837	0.965	0.690	1.351	0.185	1.263	0.894	1.783	**<0.001**	2.661	1.853	3.822	**0.025**	0.677	0.481	0.951	0.653	0.925	0.660	1.298	0.317	0.838	0.593	1.185
Yes, but I don't work from home	0.956	1.010	0.713	1.431	0.490	0.888	0.633	1.246	0.653	0.922	0.646	1.314	0.544	1.130	0.761	1.677	**0.033**	0.685	0.483	0.970	0.401	0.862	0.611	1.218	0.424	0.869	0.616	1.226
No		1				1				1				1				1				1				1		
**Area of residence**																												
Urban	**0.021**	1.385	1.050	1.827	**0.042**	1.334	1.011	1.760	**0.001**	1.593	1.198	2.118	0.053	1.373	0.996	1.892	0.465	1.109	0.840	1.466	**0.015**	1.413	1.070	1.865	**<0.001**	1.738	1.316	2.296
Semi-urban	**0.038**	1.417	1.020	1.969	0.210	1.234	0.888	1.715	**0.001**	1.722	1.233	2.406	**0.014**	1.593	1.099	2.310	0.613	1.089	0.782	1.517	0.419	1.143	0.826	1.582	**0.007**	1.563	1.128	2.166
Rural (ref.)		1				1				1				1				1				1				1		
**Household composition**																												
Alone	0.220	0.830	0.617	1.118	0.769	0.957	0.714	1.283	0.874	1.025	0.759	1.383	0.719	1.063	0.761	1.485	0.815	0.965	0.715	1.302	0.749	1.049	0.781	1.410	**0.040**	0.733	0.545	0.986
With children under 18 years of age	0.082	1.344	0.963	1.876	0.301	1.193	0.854	1.664	**0.007**	1.589	1.135	2.225	0.891	0.975	0.682	1.394	0.059	1.378	0.988	1.921	0.147	1.277	0.918	1.778	**0.006**	1.599	1.145	2.233
With people over 65 years and/or with chronic disease	0.880	1.028	0.716	1.476	0.960	0.991	0.692	1.418	0.055	1.421	0.993	2.034	0.587	1.121	0.742	1.695	0.463	1.145	0.798	1.644	0.521	1.125	0.785	1.610	0.891	0.975	0.681	1.397
None of the above (ref.)		1				1				1				1				1				1				1		
**Income**																												
Higher income group	0.219	1.598	0.757	3.371	**0.014**	2.462	1.203	5.039	0.684	0.864	0.428	1.745	0.514	1.291	0.599	2.778	**0.038**	2.155	1.044	4.450	**0.020**	2.356	1.143	4.857	**<0.001**	4.319	2.035	9.168
Higher-middle income group	0.753	0.923	0.560	1.521	0.691	1.105	0.675	1.808	**0.039**	0.586	0.352	0.974	0.678	0.889	0.510	1.549	0.068	1.607	0.966	2.675	0.971	0.991	0.605	1.624	0.359	1.269	0.763	2.111
Middle income group	0.396	1.188	0.798	1.767	0.942	0.986	0.667	1.456	0.061	0.679	0.453	1.017	0.893	0.969	0.613	1.531	**0.012**	1.690	1.121	2.546	0.237	0.787	0.530	1.170	0.170	1.324	0.887	1.976
Lower-middle income group	0.204	1.300	0.867	1.948	0.298	1.233	0.831	1.832	0.293	0.802	0.532	1.210	0.393	0.813	0.506	1.306	0.090	1.433	0.945	2.173	0.714	1.078	0.721	1.612	0.163	1.336	0.889	2.009
Lower income group (ref.)		1				1				1				1				1				1				1		
**Financial situation over last 3 years**																												
Improved	0.221	1.988	0.662	5.967	0.744	1.196	0.409	3.498	0.414	1.558	0.537	4.520	0.720	1.219	0.413	3.597	0.165	2.036	0.747	5.548	0.334	1.665	0.592	4.680	0.751	1.199	0.391	3.678
Remained the same	0.431	1.537	0.527	4.484	0.832	1.119	0.395	3.174	0.577	1.343	0.477	3.782	0.744	1.192	0.416	3.419	0.285	1.701	0.642	4.506	0.488	1.425	0.523	3.883	0.948	0.964	0.325	2.865
Worsened	0.469	1.487	0.508	4.359	0.894	1.073	0.377	3.057	0.581	1.341	0.473	3.796	0.797	1.149	0.398	3.318	0.244	1.791	0.672	4.773	0.377	1.576	0.575	4.323	0.979	0.985	0.331	2.938
I don't know (ref.)		1				1				1				1				1				1				1		
**Age**	0.264	1.005	0.996	1.013	0.760	0.999	0.990	1.007	**0.049**	1.008	1.000	1.017	**0.004**	0.986	0.977	0.996	0.968	1.000	0.992	1.008	0.075	1.008	0.999	1.016	0.812	1.001	0.993	1.009
**Trust**	**<0.001**	1.094	1.066	1.123	**<0.001**	1.080	1.051	1.108	**<0.001**	1.062	1.034	1.090	**<0.001**	1.063	1.032	1.095	**<0.001**	1.069	1.041	1.098	**<0.001**	1.098	1.069	1.127	**<0.001**	1.071	1.043	1.099
**Knowledge**	0.425	0.984	0.945	1.024	0.524	0.987	0.949	1.027	**0.012**	0.949	0.910	0.989	**0.002**	0.933	0.893	0.975	**0.016**	1.051	1.009	1.094	0.547	0.988	0.949	1.028	**<0.001**	0.925	0.889	0.963

Note: OR = odds ratio; CI = confidence interval. Bold values indicate statistical significance ($p < .05$). Reference categories are indicated by "ref.". b = Set to zero because this parameter is redundant

Table S4. Descriptive statistics of UV-light technology versus existing measures

F3. Researchers have developed a prototype machine that deactivates the virus using UV light. Would you rather:

		Gender		Age					Region				Education						Income strata				
	**Total**	Male	Female	18-29 Y.	30-39 Y.	40-49 Y.	50-59 Y.	60+ Y.	North	West	South	East	Secondary school certificate	High school diploma	Vocational training	Bachelor's degree	Master's degree	Doctorate degree	Higher income group	Highermiddle income group	Middle income group	Lowermiddle income group	Lower income group
**Germany**	**1000** ***100,0%***	489 *100,0%*	511 *100,0%*	166 *100,0%*	153 *100,0%*	150 *100,0%*	194 *100,0%*	337 *100,0%*	161 *100,0%*	351 *100,0%*	290 *100,0%*	198 *100,0%*	157 *100,0%*	153 *100,0%*	389 *100,0%*	141 *100,0%*	144 *100,0%*	16 *100,0%*	48 *100,0%*	141 *100,0%*	421 *100,0%*	267 *100,0%*	123 *100,0%*
implement the prototype machine in classrooms to prevent any school closings?	**761** ***76,1%***	366 *74,8%*	395 *77,3%*	122 *73,5%*	92 *60,1%*	111 *74,0%*	154 *79,4%*	282 *83,7%*	113 *70,2%*	271 *77,2%*	219 *75,5%*	158 *79,8%*	108 *68,8%*	110 *71,9%*	311 *79,9%*	103 *73,0%*	115 *79,9%*	14 *87,5%*	23 *47,9%*	111 *78,7%*	319 *75,8%*	213 *79,8%*	95 *77,2%*
apply established measures such as masks or school closings instead of new technologies?	**239** ***23,9%***	123 *25,2%*	116 *22,7%*	44 *26,5%*	61 *39,9%*	39 *26,0%*	40 *20,6%*	55 *16,3%*	48 *29,8%*	80 *22,8%*	71 *24,5%*	40 *20,2%*	49 *31,2%*	43 *28,1%*	78 *20,1%*	38 *27,0%*	29 *20,1%*	2 *12,5%*	25 *52,1%*	30 *21,3%*	102 *24,2%*	54 *20,2%*	28 *22,8%*

F3. Researchers have developed a prototype machine that deactivates the virus using UV light. Would you rather:

		Gender		Age					Region						Education										Income strata				
	**Total**	Male	Female	18-29 Y.	30-39 Y.	40-49 Y.	50-59 Y.	60+ Y.	Central	East	North	NorthEast	South	West	Literate without formal education	Below primary	Primary	Middle	Secondary	Higher secondary / intermediate	Nontechnical diploma or certificate (not equal to degree)	Technical diploma or certificate (not equal to degree)	Graduate	Postgraduate and above	Higher income group	Higher middle income group	Middle income group	Lower middle income group	Lower income group
**India**	**1000** ***100,0%***	510 *100,0%*	490 *100,0%*	346 *100,0%*	237 *100,0%*	166 *100,0%*	129 *100,0%*	122 *100,0%*	90 *100,0%*	250 *100,0%*	140 *100,0%*	40 *100,0%*	250 *100,0%*	230 *100,0%*	4 *100,0%*	3 *100,0%*	9 *100,0%*	15 *100,0%*	25 *100,0%*	77 *100,0%*	24 *100,0%*	51 *100,0%*	450 *100,0%*	342 *100,0%*	72 *100,0%*	281 *100,0%*	521 *100,0%*	108 *100,0%*	18 *100,0%*
implement the prototype machine in classrooms to prevent any school closings?	**566** ***56,6%***	280 *54,9%*	286 *58,4%*	188 *54,3%*	139 *58,6%*	94 *56,6%*	75 *58,1%*	70 *57,4%*	52 *57,8%*	141 *56,4%*	77 *55,0%*	24 *60,0%*	142 *56,8%*	130 *56,5%*	2 *50,0%*	3 *100,0%*	6 *66,7%*	6 *40,0%*	12 *48,0%*	44 *57,1%*	11 *45,8%*	27 *52,9%*	259 *57,6%*	196 *57,3%*	36 *50,0%*	160 *56,9%*	290 *55,7%*	69 *63,9%*	11 *61,1%*

apply established measures such as masks or school closings instead of new technologies?	**434**	230	204	158	98	72	54	52	38	109	63	16	108	100	2	-	3	9	13	33	13	24	191	146	36	121	231	39	7
	43,4%	*45,1%*	*41,6%*	*45,7%*	*41,4%*	*43,4%*	*41,9%*	*42,6%*	*42,2%*	*43,6%*	*45,0%*	*40,0%*	*43,2%*	*43,5%*	*50,0%*	-	*33,3%*	*60,0%*	*52,0%*	*42,9%*	*54,2%*	*47,1%*	*42,4%*	*42,7%*	*50,0%*	*43,1%*	*44,3%*	*36,1%*	*38,9%*
